# Butyrophilin-like 9 expression is associated with outcome in lung adenocarcinoma

**DOI:** 10.1186/s12885-021-08790-9

**Published:** 2021-10-11

**Authors:** Weishuang Ma, Jiaming Liang, Junjian Mo, Siyuan Zhang, Ningdong Hu, Dongbo Tian, Zisheng Chen

**Affiliations:** 1 Department of Respiratory Medicine, The Sixth Affiliated Hospital of Guangzhou Medical University Qingyuan People’s Hospital, Qingyuan, China; 2 Zhouxin Community Health Service, Qingcheng District, Qingyuan, China; 3 State Key Laboratory of Respiratory Disease, The First Affiliated Hospital of Guangzhou Medical University, National Clinical Research Center for Respiratory Disease, Guangzhou, China; 4 Department of Thoracic Surgery, The Sixth Affiliated Hospital of Guangzhou Medical University, Qingyuan People’s Hospital, Qingyuan, China

**Keywords:** Butyrophilin-like 9, B cells, Dendritic cells, Prognosis, Lung adenocarcinoma

## Abstract

**Background:**

Lung adenocarcinoma (LUAD) is the most prevalent non-small cell lung cancer (NSCLC). Patients with LUAD have a poor 5-year survival rate. The use of immune checkpoint inhibitors (ICIs) for the treatment of LUAD has been on the rise in the past decade. This study explored the prognostic role of butyrophilin-like 9 (*BTNL9*) in LUAD.

**Methods:**

Gene expression profile of buytrophilins (*BTNs)* was determined using the GEPIA database. The effect of *BTNL9* on the survival of LUAD patients was assessed using Kaplan-Meier plotter and OncoLnc. Correlation between *BTNL9* expression and tumor-infiltrating immune cells (TILs) was explored using TIMER and GEPIA databases. Further, the relationship between *BTNL9* expression and drug response was evaluated using CARE. Besides, construction and evaluation of nomogram based on *BTNL9* expression and TNM stage.

**Results:**

*BTNL9* expression was downregulated in LUAD and was associated with a poor probability of 1, 3, 5-years overall survival (OS). In addition, *BTNL9* expression was regulated at epigenetic and post-transcriptional modification levels. Moreover, *BTNL9* expression was significantly positively correlated with ImmuneScore and ESTIMATEScore. Furthermore, *BTNL9* expression was positively associated with infiltration levels of B cells, CD4^+^ T cells, and macrophages. Kaplan-Meier analysis showed that *BTNL9* expression in B cells and dendritic cells (DCs) was significantly associated with OS. *BTNL9* expression was significantly positively correlated with CARE scores.

**Conclusions:**

These findings show that *BTNL9* is a potential prognostic biomarker for LUAD. Low *BTNL9* expression levels associated with low infiltration levels of naïve B cells, and DCs in the tumor microenvironment are unfavorable for OS in LUAD patients.

**Supplementary Information:**

The online version contains supplementary material available at 10.1186/s12885-021-08790-9.

## Background

Lung cancer is the most common cancer and the leading cause of cancer-related deaths globally and in China [1, 2]. Although the 5-year survival rate has increased over the past four decades, the OS is poor (5.6–20.6%) [3]. Immunotherapies have significantly improved cancer treatment during the past decade. For example, pembrolizumab used to treat naive advanced non–small-cell lung cancer (NSCLC) shows a 5-year survival rate of 23.2 and 29.6% in patients with a PD-L1 tumor proportion score ≥ of 50% [4]. Immune checkpoint inhibitors (ICIs) block immune checkpoint signaling, thus alleviating antitumor immunity, and significantly improving five year-OS in NSCLC.

PD-1/PD-L1 is the most widely used ICIs, whereas other immune checkpoints, such as LAG-3, TIGIT, TIM-3, and CTLA-4, are currently under development [5]. However, the biological role of immune checkpoint buytrophilins *(BTNs)* [6] in the regulation of NSCLC physiology and its underlying molecular mechanism remains to be fully elucidated. *BTNs*, including butyrophilin (*BTN*) and butyrophilin-like (*BTLN*), are related to the B7 family of co-stimulatory molecules. This family plays a significant role in T cell suppression, regulating epithelial cell and T cell interplays [7]. Human *BTN* genes are located in the MHC class I domain of the short arm of chromosome 6 (6p22.1). Human *BTN* genes are grouped into three subfamilies, which form phylogenetically related groups, including *BTN1*, *BTN2*, and *BTN3*. *BTN1A1* belongs to the *BTN1* subfamily, *BTN2A1, BTN2A2*, and *BTN2A3* (*BTN2A3P*) belong to the *BTN2* subfamily, whereas *BTN3A1*, *BTN3A2*, and *BTN3A3* belong to the *BTN3* subfamily. Moreover, butyrophilin-like proteins (*BTNL*: *BTNL2*, *BTNL3*, *BTNL8*, *BTNL9*, and *BTNL10*) and SKINT-like (*SKINTL*) are classified in the family of *BTNs* [7–9].

**Fig. 1:**
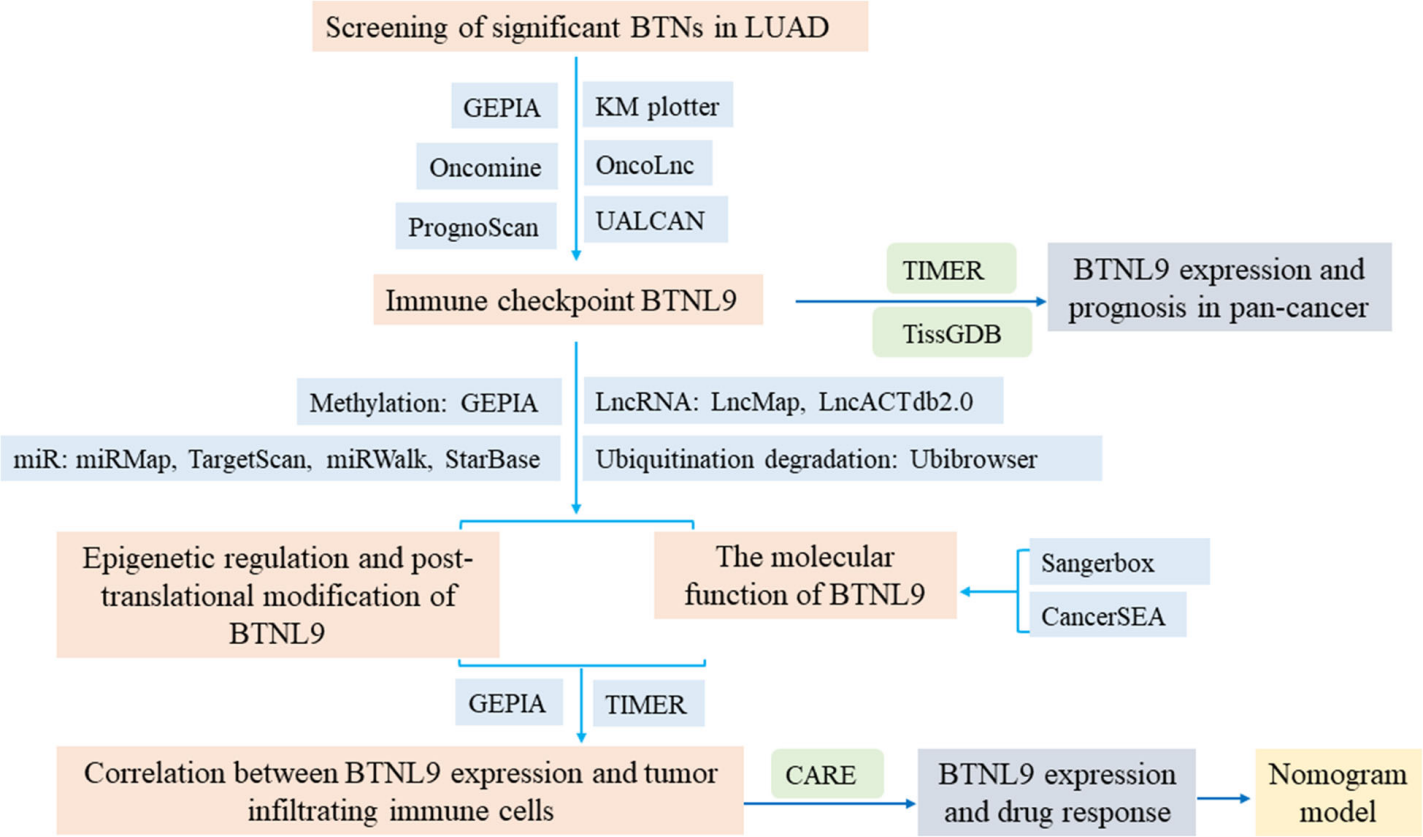
A flow chart of the study design

Lung adenocarcinoma (LUAD) has been the most prevalent histopathological subtype of NSCLC in China since 2014 [10]. In this study, we explored the relationship between the expression level of *BTN*s and LUAD prognosis. Significant survival-related *BTNs* were screened using GEPIA [11]. Datasets used for analysis in this study were retrieved from Gene Expression Omnibus [12], TIMER [13], KM plotter [14], UALCAN [15], OncoLnc [16], Oncomine [17], TissGDB [18] databases. The findings from this study provide information on the relationship between immune checkpoint *BTNL9* and tumor immune response. These findings show that *BTNL9* can be used for the prognosis and development of immunotherapy for LUAD. A flow chart of the study design is shown in Fig. 1.

## Methods

### Determination of expression profiles of *BTNs* genes

Expression profiles of *BTNs* in LUAD were determined using the GEPIA database [19]. Default settings were used with |Log2FC| > 1 and *p*-value Cutoff < 0.01 used as the cutoff criteria to determine differentially expressed genes. Log2(TPM + 1) was transformed for gene expression profile; jitter size was set at 0.4 for the plot. Notably, TCGA and GTEx datasets were included in the analysis. mRNA expression profile of *BTNL9* in different tumor types was determined using the Oncomine database [17] using default settings with a *P*-value of 0.01, a fold change of 1.5, and a top 10% gene ranking.

### Survival analysis

Median gene expression was used as the cutoff point for survival analysis. Survival analysis of *BTNL9* was performed using three online databases, including KM plotter [14], UALCAN [15], and OncoLnc databases [16]. TissGDB is a tissue-specific gene annotation database in cancer [18]. Forest plot were generated using Cox proportional hazard ratio (HR), and overall survival (OS) and relapse-free survival (RFS) of 28 cancer types were performed using 95% CI.

### Estimation of infiltration level of immune cell type and correlation with *BTNL9*

TIMER is a comprehensive resource for systematically analyzing immune infiltration in diverse cancer types based on the TCGA dataset [13, 20]. The Gene_DE module from TIMER was used to calculate *BTNL9* mRNA expression in cross-carcinoma (*: *P*-value < 0.05; **: *P*-value < 0.01; ***: *P*-value < 0.001). The expression profile of *BTNL9* in LUAD and its correlation with six immune infiltration cells, including B cells, CD4+ T cells, CD8+ T cells, macrophages, neutrophils, and DCs, were analyzed using Gene and Survival module in TIMER. Gene_Corr module was used to determine the correlation between *BTNL9* expression and B and DC cells [21]. The immune score and stromal score of each TCGA tumor sample were estimated using Sangerbox (http://sangerbox.com/Index).

### Predicting binding of miRNA and lncRNA to *BTNL9*

miRMap [22], TargetScan [23], and miRWalk [24] were used to predict miRNAs that can bind to *BTNL9*. Predicted miRNAs obtained from the three databases were further verified using the starBase database [25].

### Gene set enrichment analysis (GSEA) of *BTNL9* high and low expression groups

Sangerbox is a tool developed by Hangzhou Mugu Technology Co., Ltd. GSEA was used to perform KEGG and HALLMARK pathway analysis for the *BTNL9* high and low expression groups based on the TCGA database.

### Estimating drug response for LUAD

Computational Analysis of Resistance (CARE) is a software that uses compound screening data to identify genome-scale biomarkers for targeted therapeutic strategies. Pearson correlation analysis between the gene expression profile of the cancer sample and the CARE scoring vector was used to group the patient as a responder or a non-responder [26].

### Construction and evaluation of nomogram

We acquired TCGA LUAD RNA-seq data from the University of California, Santa Cruz (UCSC) Xena Browser (https://xenabrowser.net/). After screening, samples with missing clinical data and 0 days overall survival time were excluded, and a total of 501 samples were included. Next, we randomly divided the TCGA-LUAD cohort (*n* = 501) in a 7 to 3 ratio into a training (*n* = 352) and testing dataset (*n* = 149). We performed the R package “rms” to construct a nomogram based on the TNM stage and expression profile of *BTNL9* using the training dataset. To evaluate the usefulness of the nomogram, the R package “ROCsurvival” was used to construct ROC for the prediction of the 1-, 3- and 5- year OS. The R package “ggDCA” was executed to create a decision analysis curve to evaluate the clinical utility of the nomogram. Finally, R package “rms” was applied to perform a calibration curve to evaluate the precision for predicting 1-, 3- and 5-year OS prediction of the LUAD cohort.

### Statistical analysis

The relationship between *BTNL9* expression and single cancer cell biological behavior of LUAD was determined using Pearson correlation analysis and Spearman’s correlation analysis of the correlation between *BTNL9* and tumor mutation burden (TMB). In all the studies, *P* < 0.05 was considered statistically significant.

## Results

### The high expression level of *BTNL9* was associated with favorable survival of LUAD

**Fig. 2:**
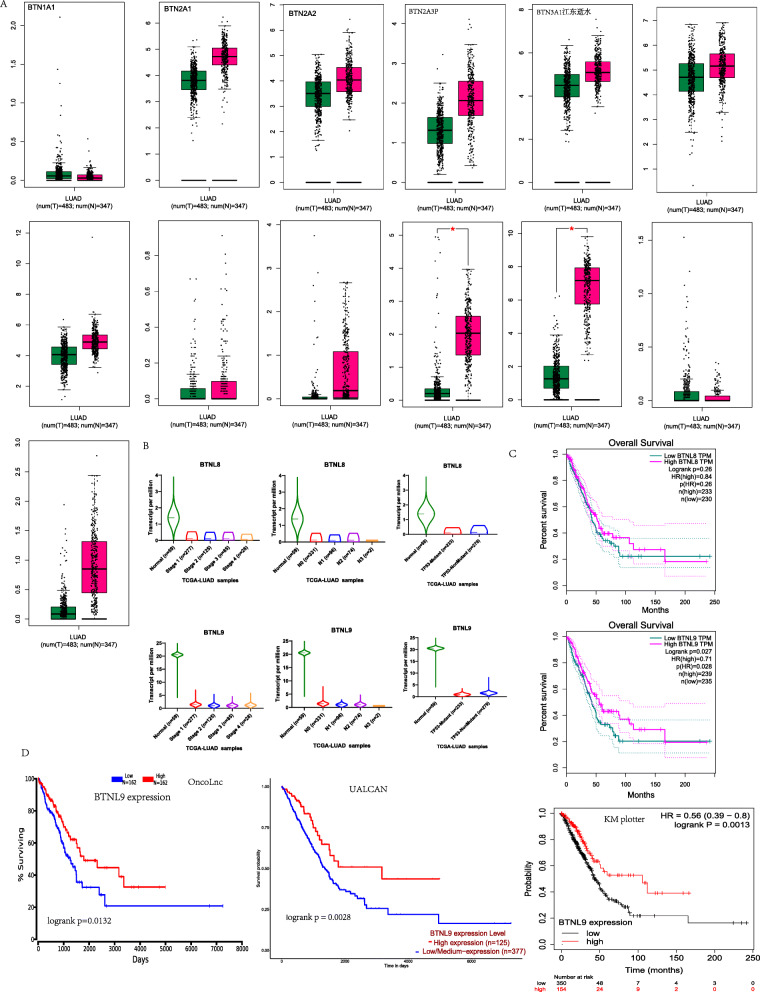
*BTNL9* Expression and prognosis in LUAD. (**A**) *BTNs* expression panel in LUAD tissues compared with adjacent tissues in GEPIA database. (**B**) Correlation between *BTNL8* and *BTNL9* expression and LUAD clinical stages, N stage, and *p53* mutation in the UALCAN database (*BTNL8* expression compared with LUAD clinical stages (Normal-vs-Stage1, *P* = 1.01E-04; Normal-vs-Stage2, *P* = 7.08E-03; Normal-vs-Stage3, *P* = 1.36E-04; Normal-vs-Stage4, *P* = 1.78E-08), N stage (Normal-vs-N0, *P* = 6.56E-04; Normal-vs-N1, *P* = 8.27E-10; Normal-vs-N2, *P* = 5.96E-04; Normal-vs-N3, *P* = 1.97E-10; N0-vs-N1, *P* = 3.27E-03; N0-vs-N3, *P* = 6.79E-04), and *p53* status (Normal-vs-T P53-Mutant, *P* = 1.27E-06; Normal-vs-T P53-NonMutant, *P* = 1.80E-03). *BTNL9* expression comparison with LUAD clinical stages (Normal-vs-Stage1, *P* = 1.21E-12; Normal-vs-Stage2, *P* = 3.60E-13; Normal-vs-Stage3, *P* = 2.40E-12; Normal-vs-Stage4, *P* = 2.25E-12), N stage (Normal-vs-N0, *P* = 1.08E-12; Normal-vs-N1, *P* = 1.85E-12; Normal-vs-N2, *P* = 1.02E-12; Normal-vs-N3, *P* = 1.67E-12; N0-vs-N1, P = 1.02E-03; N2-vs-N3, *P* = 3.01E-02), and P53 status (Normal-vs-T P53-Mutant, P = 1.85E-12; Normal-vs-T P53-NonMutant, *P* = 4.10E-12; T P53-Mutant-vs-T P53-NonMutant, *P* = 1.77E-04)). (**C**) Correlation between *BTNL8* and *BTNL9* expression with overall survival of LUAD using GEPIA database. (**D**) Correlation between *BTNL9* expression and LUAD overall survival using OncoLnc, UALCAN, and KM plotter databases. *: *P*-value < 0.05; **: *P*-value < 0.01; ***: *P*-value < 0.001

Gene expression profile of *BTNs*, including *BTN1A1*, *BTN2A1*, *BTN2A2*, *BTN2A3P*, *BTN3A1*, *BTN3A2*, *BTN3A3*, *BTNL2*, *BTNL3*, *BTNL8*, *BTNL9*, *BTNL10,* and *SKINTL* was evaluated in normal and tumor lung tissues (Fig. 2A). Analysis showed that expression levels of *BTNL8* and *BTNL9* were significantly lower in tumor tissues compared with that of normal tissues (Fig. 2A). Furthermore, the expression level of *BTNL9* was significantly negatively correlated with the clinical stage, lymph node metastasis stage, and *p53* mutation. Concurrently, the expression level of *BTNL8* was significantly negatively correlated with the clinical stage and N stage (Fig. 2B). However, survival analysis showed that *BTNL8* was not significantly correlated with OS, whereas *BTNL9* was significantly correlated with OS in LUAD (Fig. 2C). Validation of the prognosis value of *BTNL9* in LUAD cohorts using PrognoScan [27] showed that *BTNL9* expression was significantly correlated with RFS and OS in the GSE31210 dataset (*n* = 204). In addition, the expression level of *BTNL9* was significantly associated with OS in GSE3141 cohort (*n* = 111) (Supplementary Table 1). Analysis using OncoLnc, UALCAN, and KM plotter showed that high expression of *BTNL9* is significantly associated with better OS in LUAD (Fig. 2D). Although the two survival curves crossover occurred after 150 months (Fig. 2C), it is well beyond 5-years (60 months), and survival curves in the verification databases didn’t show crossover. Thus, we considered the results in this study reliable and stable. These findings imply that *BTNL9* is a critical immune checkpoint of *BTN*s in LUAD.

Furthermore, the clinical distribution of *BTNL9* was explored. TCGA-LUAD dataset was divided into the high and low groups based on the median gene expression level of *BTNL9*. The clinical characteristics, including age, gender, race, TNM staging, ECOG score, EGFR mutations, KRAS mutations, and radiotherapy information, were compared between the two groups. Analysis showed no significant difference in these clinical characteristics between the two groups (data not shown).

### Pan- cancer gene expression and prognostic value of *BTNL9*

**Fig. 3:**
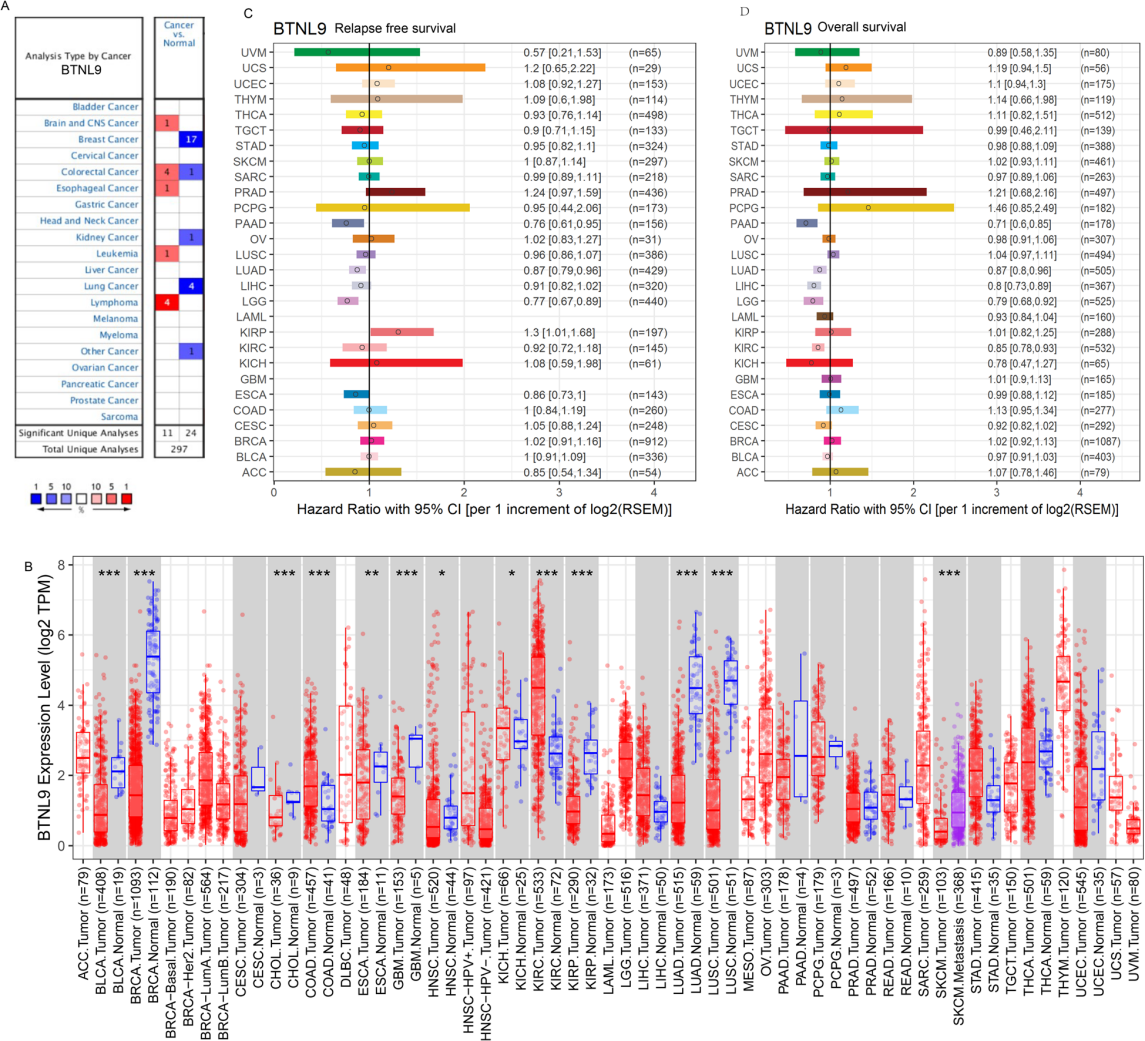
mRNA expression level of *BTNL9* in pan-cancer and hazard ratio of relapse-free survival and overall survival in LUAD patients. (**A**) Increased or decreased expression of *BTNL9* in various tumors compared with adjacent tissues using the Oncomine database. (**B**) *BTNL9* expression in pan-cancer analysis using TCGA dataset analyzed by TIMER (*P < 0.05, **P < 0.01, ***P < 0.001). (**C**) *BTNL9* expression and hazard ratio of relapse-free survival, and (**D**) overall survival using the TissGDB database

To further understand *BTNL9* expression in pan-cancer, analysis of the dataset was performed using the Oncomine database. The findings showed that *BTNL9* expression level was significantly lower in breast cancer, one colon cancer cohort, lung cancer, kidney cancer, and crabtree uterus cancer than normal tissues. However, *BTNL9* expression was significantly higher in the brain and CNS cancer, colorectal cancer, esophageal cancer, leukemia, and lymphoma, than in normal tissue (Fig. 3A and Supplementary Table 2). Further, the *BTNL9* expression profile was explored using TCGA RNA sequencing data (TIMER). The *BTNL9* expression level was significantly downregulated in Bladder Urothelial Carcinoma (BLCA), Breast invasive carcinoma (BRCA), Cholangiocarcinoma (CHOL), Esophageal carcinoma (ESCA), Glioblastoma multiforme (GBM), Head and Neck squamous cell carcinoma (HNSC), Kidney renal papillary cell carcinoma (KIRP), LUAD, and LUSC compared with normal tissues. In contrast, *BTNL9* was significantly increased in Colon adenocarcinoma (COAD), Kidney Chromophobe (KICH), and Kidney renal clear cell carcinoma (KIRC) compared with normal tissues (Fig. 3B). Analysis using the TissGDB database gave a correlation coefficient of *BTNL9* expression with LUAD’s RFS HR of 0.87 [95% CI (0.79, 0.96)], and that for the correlation between *BTNL9* expression and OS HR was 0.87 [95% CI (0.8,0.96)] (Fig. 3C, D).

### Upstream and downstream regulatory network of *BTNL9*

**Fig. 4:**
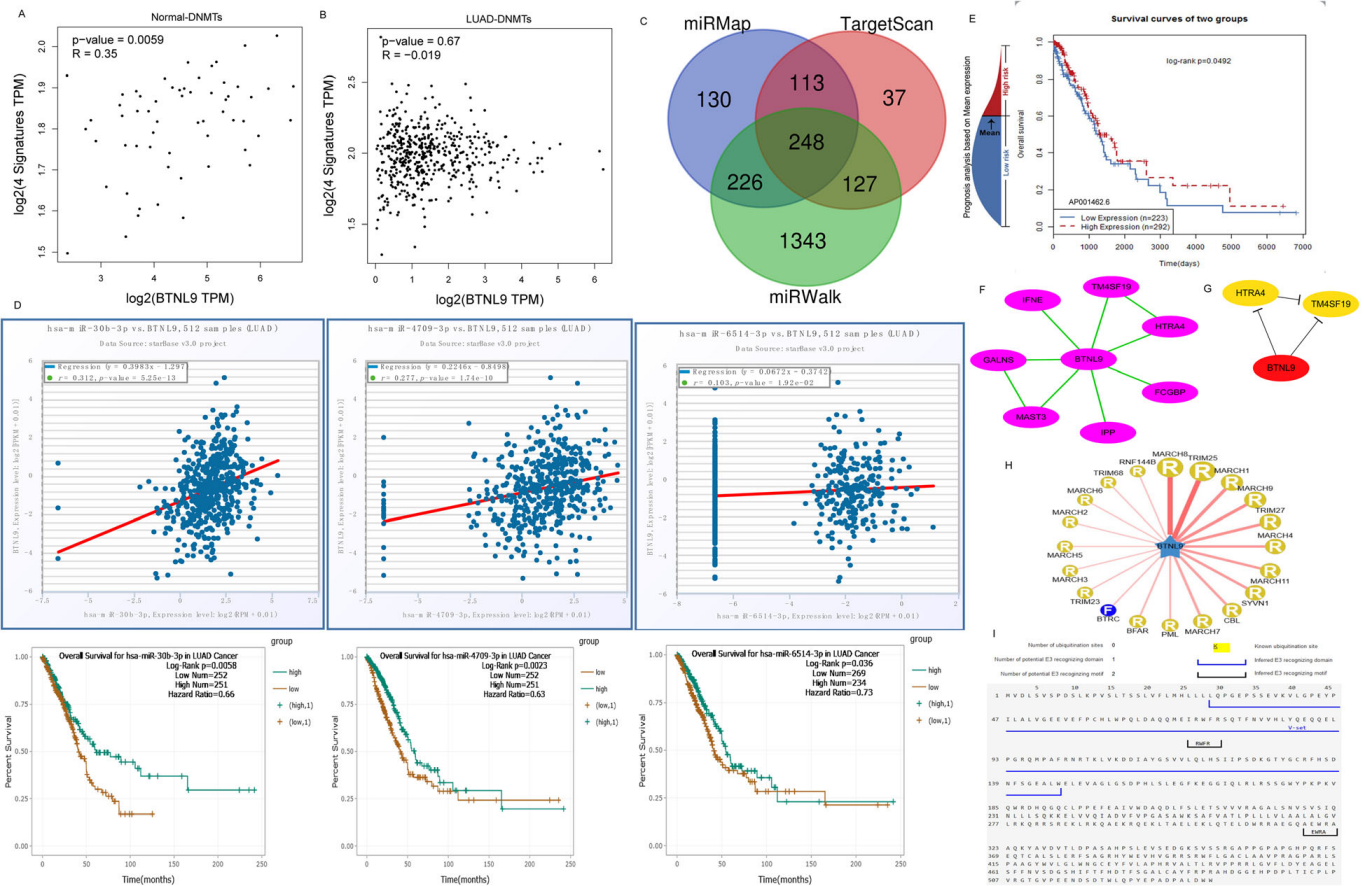
Epigenetic regulation and post-translation modulation network of *BTNL9* in LUAD. (**A**, **B**) Correlation between *BTNL9* expression and methyltransferases (DNMTs) such as DNMT1, DNMT2, DNMT3A, and DNMT3B in LUAD and adjacent tissues using GEPIA. (**C**) Predicted miRNAs that bind to *BTNL9* using miRMap, TargetScan, and miRWalk databases presented as a Venn diagram. (**D**) Overlapping 248 miRNAs verified using the StarBase database, hsa-miR-30b-3p, hsa-miR- 4709-3p, and hsa-miR-6514-3p were screened. (**E**) Predicted LncRNAs that bind to *BTNL9* were predicted using the LncMap database. AP001462.6 was verified and screened using the LncACTdb2.0 database. (**F**, **G**) *BTNL9* interacting proteins were identified using the STRING database and edited and visualized using Cytoscape software (V3.7.2). Hub genes were screened using the cytoHubba module in Cytoscape. (**H**, **I**) Ubibrowser database predicts that the substrate *BTNL9* can be bound by E3 (MARCH8) ligases, with one potential E3 recognizing domain and two potential E3 identifying motifs

A previous study reports that the expression level of *BTNL9* in LUAD is significantly lower than that in normal tissues. DNA methylation is a biological process through which methyl groups are added to DNA molecules by Methyltransferases (DNMTs). DNA methylation of a gene promoter region functions by inhibiting gene transcription. Correlation analysis between *BTNL9* expression and DNA methylation marker DNMTs (including DNMT1, DNMT2, DNMT3A, DNMT3B) was conducted using the GEPIA tool. Analysis showed that expression of *BTNL9* in normal lung tissue was positively correlated with DNMTs (r = 0.35, *P* = 0.0059); however, there was no correlation with DNMTs in LUAD (r = − 0.019, *P* = 0.67) (Fig. 4A, B). These findings show that DNA methylation may be involved in the pathogenesis of LUAD. To further explore the upstream regulation mechanisms of the *BTNL9* expression, miRNAs that bind to *BTNL9* were predicted by using miRMap [22], TargetScan [23], and miRWalk [24] databases. A total of 248 miRNAs common predicted miRNAs from the three databases were obtained (Fig. 4C) and used starBase [25] to validate the predicted binding miRNAs. Analysis showed that, hsa-miR-30b-3p, hsa-miR-4709-3p and hsa-miR-6514-3p were significantly positively correlated with *BTNL9* expression (r = 0.312, *P* = 5.25E-13, r = 0.277, *P* = 1.74E-10, and r = 0.103, *P* = 0.02, respectively, Fig. 4D). In addition, the three miRNAs were highly expressed and significantly correlated with higher OS of LUAD patients (HR = 0.66, *P* = 0.0058, HR = 0.63, *P* = 0.0023, and HR = 0.73, *P* = 0.036, respectively) (Fig. 4D). Although the two survival curves of hsa-miR-4709-3p and hsa-miR-6514-3p crossover occurred after 100 months, it is well beyond 5-years (60 months). Thus, we considered the results in this study reliable. Furthermore, 18 lncRNAs were predicted to bind to *BTNL9* using LncMap [28] database (Supplementary Table 3). These findings were verified using LncACTdb2.0 [29] database. LncRNA AP001462.6 was predicted to bind to *BTNL9*, and the high expression level of AP001462.6 was significantly correlated with a high OS of LUAD patients (*P* = 0.049) (Fig. 4E).

Moreover, proteins implicated in binding *BTNL9* were analyzed using the STRING [30] database. Analysis showed a total of 7 proteins that bind to *BTNL9* (Fig. 4F). The top 2 binding proteins, including HTRA4 and TM4SF19, were predicted using the cytoHubba module of Cytoscape [31] (Fig. 4G). HTRA4 gene encodes a member of the HtrA protease family. HTRA4 plays a role as a secreted oligomeric chaperone protease to degrade misfolded secretory proteins [19]. We hypothesized that low expression of *BTNL9* in LUAD might be related to degradation through ubiquitination. Analysis using Ubibrowser [32] database showed that E3 (MARCH8) ligases could bind the substrate *BTNL9* (Supplementary Table 4). In addition, *BTNL9* has a potential E3 recognizing domain and two potential E3 identifying motifs (Fig. 4H, I).

### Low expression of *BTNL9* significantly enriches proteasome and increases cancer malignancy

**Fig. 5:**
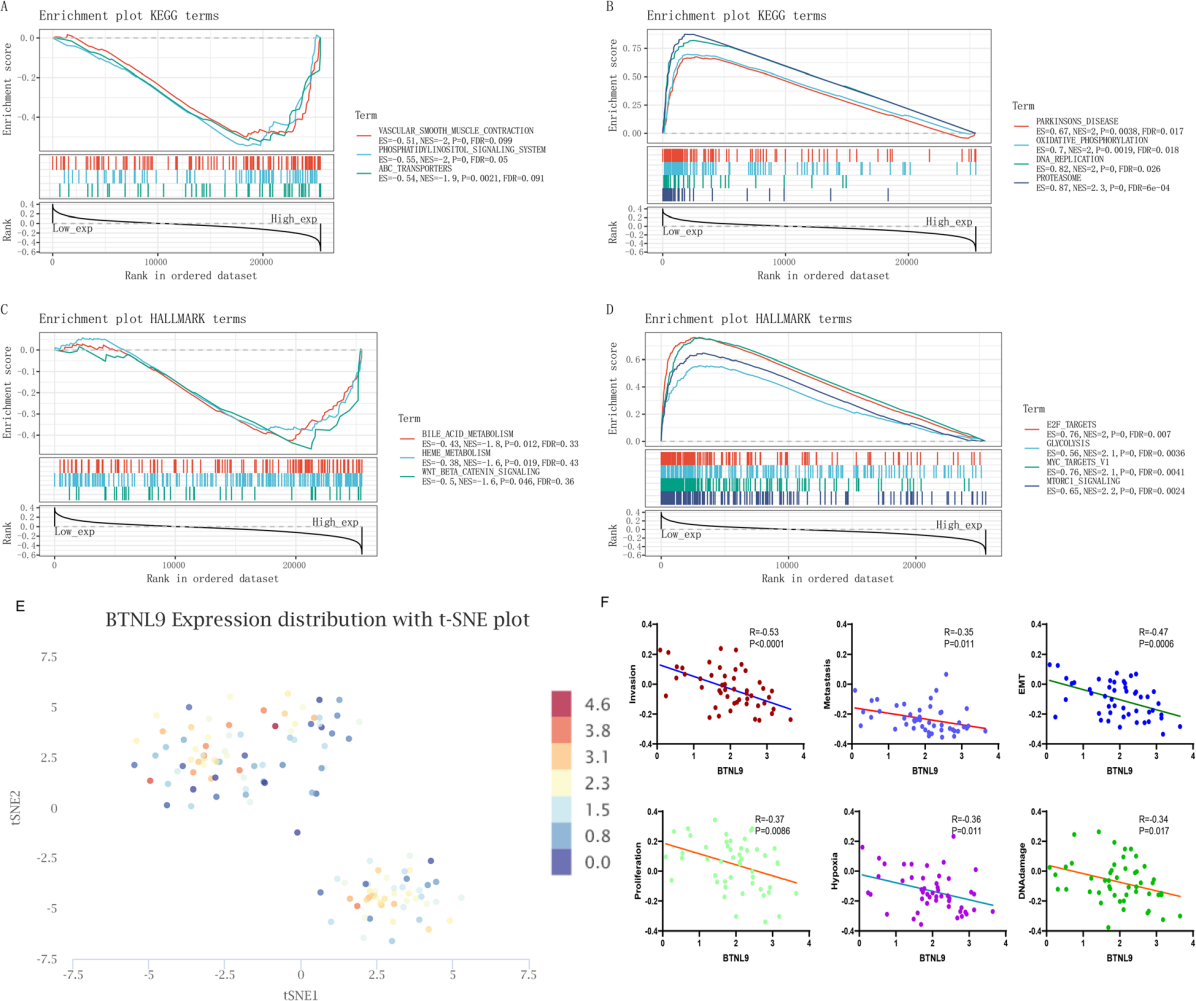
Low expression of *BTNL9* significantly enriches proteasome and promotes tumor malignancy in LUAD. (**A**, **B**, **C**, **D**) High and low *BTNL9* expression groups were presented using the Sangerbox tool and GSEA for KEGG and HALLMARK pathways. (**E**) the t-SNE plot shows scRNA analysis of *BTNL9* expression, and (**F**) molecular function of *BTNL9* in LUAD using CancerSEA database shows that *BTNL9* is significantly negatively correlated with tumor invasion, metastasis, EMT, proliferation, hypoxia, and DNA damage in LUAD

Gene Set Enrichment Analysis (GSEA) analysis for KEGG and HALLMARK was performed using the Sangerbox tool to explore the two groups’ biological pathways. The findings showed that the top 3 significantly enriched KEGG pathways in the high *BTNL9* expression group were vascular smooth muscle contraction, phosphatidylinositol signaling system, and abc transporters (Fig. 5A). On the other hand, the top 4 significantly enriched KEGGs pathways in the low *BTNL9* expression group were pathways implicated in Parkinson’s disease, oxidative phosphorylation, DNA replication, and proteasome pathways (Fig. 5B). GSEA for the HALLMARK pathway showed that the top 3 pathways associated with high *BTNL9* expression were bile acid metabolism, heme metabolism, and Wnt/beta-catenin signaling pathways. Further, the top 4 pathways associated with low *BTNL9* expression were E2F targets, glycolysis, myc targets v1, and mTORC1 signaling (Fig. 5C, D). These findings imply that *BTNL9* is involved in LUAD metabolic reprogramming.

Metabolic reprogramming is a hallmark of cancer, and intrinsic and extrinsic factors contribute to various metabolic phenotypes in tumors. As cancer develops from pre-tumor lesions to local, clinically obvious malignant tumors to metastatic cancer, metabolism changes the phenotype and dependence [33]. Single-cell RNA (scRNA) analysis of LUAD using CancerSEA [34] database (Fig. 5E) showed that *BTNL9* expression is significantly negatively correlated with tumor malignant features including invasion (r = − 0.53, *P* < 0.0001), metastasis (r = − 0.35, *P* = 0.011), EMT (r = − 0.47, *P* = 0.0006), proliferation (r = − 0.37, *P* = 0.0086), Hypoxia (r = − 0.36, P = 0.011), and DNA damage (r = − 0.34, *P* = 0.017) (Fig. 5F). This finding implies that low expression of *BTNL9* is significantly associated with the malignant features of LUAD.

### Correlation between *BTNL9* and infiltrating immune cell markers

**Fig. 6:**
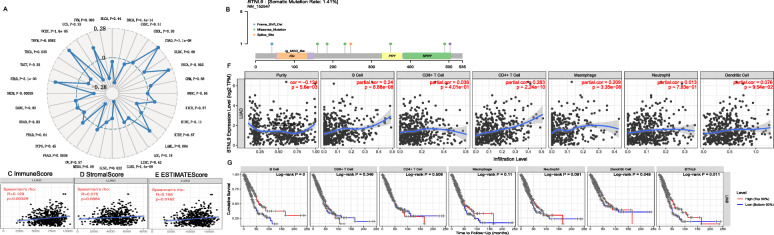
Correlation between *BTNL9* and tumor-infiltrating immune cells and OS prognosis. (**A**) Correlation analysis between *BTNL9* and TMB using Sangerbox tool, (**B**) somatic mutation pattern of *BTNL9* in LUAD, and (**C**, **D**, **E**) ESTIMATE scores. (**F**) Correlation between *BTNL9* and TME infiltrating immune cells analyzed using TIMER database, and (**G**) overall survival

Spearman’s correlation analysis of the correlation between expression of *BTNL9* and tumor mutation burden (TMB) in the TCGA LUAD cohort showed that *BTNL9* is significantly negatively correlated with TMB (*P* = 1.4E-9) (Fig. 6A). Analysis of somatic mutation pattern of *BTNL9* in LUAD using the SangerBox tool showed that the mutation frequency of *BTNL9* in LUAD was 1.41% (Fig. 6B). Genetic mutations are implicated in the tumor microenvironment (TME) [35]; therefore, the relationship between the expression of *BTNL9* and the immune score was determined using the ESTIMATE algorithm in the SangerBox tool. Analysis showed that *BTNL9* was significantly positively correlated with ImmuneScore (r = 0.129, *P* = 0.003) and ESTIMATEScore (r = 0.106, *P* = 0.016). However, the expression of *BTNL9* was not significantly correlated with StromalScore (Fig. 6C-E).

Analysis of the correlation between gene expression of *BTNL9* and infiltrating level of immune cells using TIMER [13] database showed that *BTNL9* was negatively correlated with tumor purity (r = − 0.124, *P* = 5.6E-03). On the other hand, gene expression of *BTNL9* was significantly positively correlated with B cells (r = 0.24, *P* = 8.88E-8), CD4^+^T (r = 0.283, *P* = 2.24E-10) and macrophages (r = 0.209, *P* = 3.35E-6) (Fig. 6F). Moreover, survival analysis showed that high expression of *BTNL9* in B cells (*P* = 0.000) and DC cells (*P* = 0.048) was correlated with better OS for LUAD (Fig. 6G).

A detailed analysis of TME infiltrated DC and B cells using the TIMER database showed that DC and its subtypes cDCs1 and cDCs2 [36] are associated with *BTNL9* expression before and after purity adjustment. GEPIA database analysis showed that normal lung tissue was not correlated with DC and its subtypes cDCs1 and cDCs2. However, DC and its subtypes were significantly positively correlated with LUAD (Table 1), implying that DCs regulated by *BTNL9* may participate in LUAD immune response. B cells are heterogeneous and include two subtypes: naïve B cells and plasma B cells [37]. TIMER analysis showed that total B cells and naïve B cells were significantly correlated with *BTNL9* expression before and after purity adjustment. However, plasma B cells were not associated with *BTNL9* expression before and after purity adjustment. GEPIA analysis showed that total B cells and naïve B cells were not correlated with *BTNL9* expression in normal lung tissues; however, they were significantly positively correlated with *BTNL9* expression in LUAD tissues. Plasma B cells showed no correlation with *BTNL9* in both normal tissues and LUAD tissues (Table 1), indicating that *BTNL9* may play a role in promoting naïve B cell antitumor immune response.

**Table 1:** Correlation analysis between *BTNL9* and relate gene set markers of significant innate and adaptive immunity cells in TIMER and GEPIA database

		TIMER				GEPIA			
Immune cell	Marker	None		purity		Normal		Cancer	
		Spearman’s ρ	***P*** Value	Spearman’s ρ	***P*** Value	Spearman correlation coefficient	***P*** Value	Spearman correlation coefficient	***P*** Value
**DC**	CD1C	0.36	1.65E-17	0.34	1.03E-14	−0.022	0.87	0.33	1.5E-13
	HLA-DPA1	0.23	1.64E-07	0.20	8.47E-06				
	HLA-DPB1	0.29	8.39E-12	0.27	8.03E-10				
	HLA-DQB1	0.21	1.31E-06	0.18	8.16E-05				
	HLA-DRA	0.18	4.54E-05	0.14	0.0020				
	ITGAX	0.24	1.63E-08	0.22	1.10E-06				
	NCR1	0.04	0.35980601	0.01	0.8450				
	NRP1	0.03	0.41981037	0.03	0.4973				
**cDC1s**	CD8A	0.03	0.55316497	−0.03	0.5613	−0.028	0.83	0.22	1.20E-06
	CLEC9A	0.40	1.00E-21	0.38	1.16E-18				
	XCR1	0.41	2.70E-22	0.39	7.37E-20				
**cDC2s**	CLEC12A	0.22	3.50E-07	0.18	3.61E-05	0.18	0.16	0.45	7.1E-25
	ESAM	0.51	1.01E-35	0.50	2.89E-33				
**B cell**	CD19	0.23	1.93E-07	0.20	5.30E-06	0.017	9.00E-01	0.37	8.30E-17
	FCER2	0.40	7.97E-21	0.38	2.18E-18				
	MS4A1	0.34	6.45E-16	0.33	2.59E-14				
	SDC1	0.07	0.0917	0.09	0.0523				
**Naïve B cell**	CD19	0.23	1.93E-07	0.20	5.30E-06	0.082	0.54	0.43	8.60E-23
	CD22	0.50	2.44E-34	0.51	1.10E-34				
	CD83	0.33	6.56E-15	0.31	2.09E-12				
	MS4A1	0.34	6.45E-16	0.33	2.59E-14				
	TCL1A	0.25	1.57E-08	0.21	1.31E-06				
**Plasma B cell**	CD38	0.04	0.3699	0.08	0.0746	−0.19	0.15	0.059	0.19
	TNFRSF17	0.04	0.4053	0.08	0.0814				

### High expression of *BTNL9* is associated with tyrosine kinase inhibitors response

**Fig. 7:**
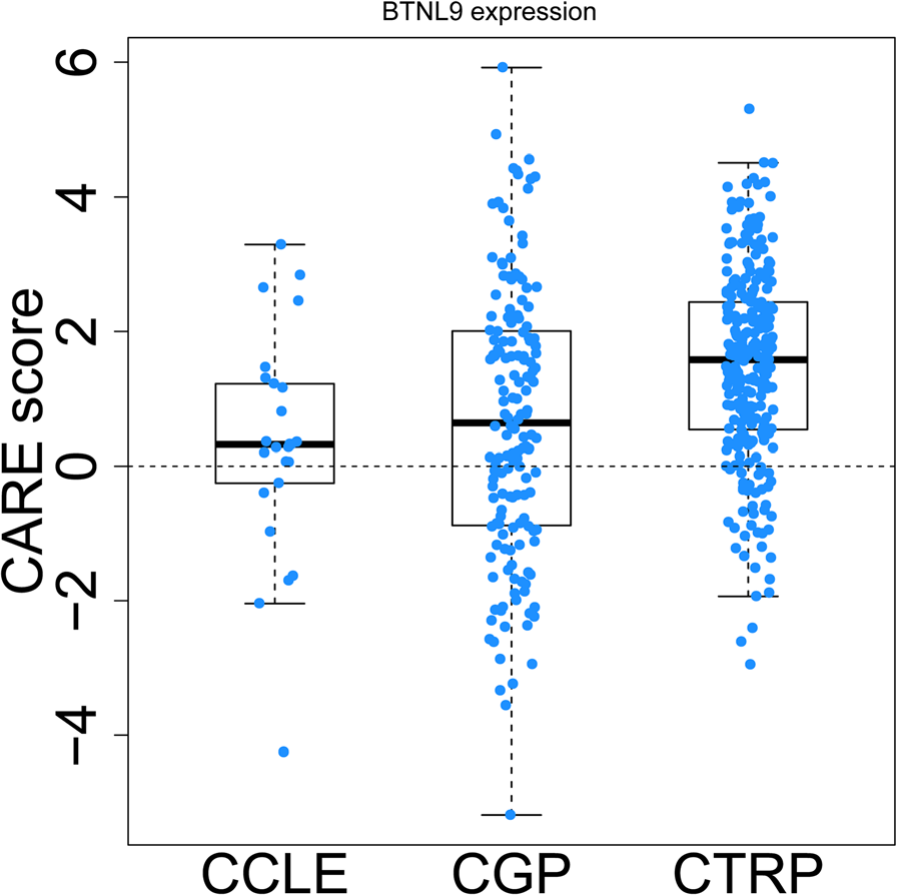
*BTNL9* expression is associated with drug response. *BTNL9* expression was significantly positively correlated with CARE scores for many compounds retrieved from Cancer Cell Line Encyclopedia (CCLE), Genomics of Drug Sensitivity in Cancer (GDSC, previously named CGP), and Cancer Therapeutics Response Portal (CTRP) databases

*BTNL9* expression was significantly positively correlated with CARE scores for several compounds retrieved from Cancer Cell Line Encyclopedia (CCLE), Genomics of Drug Sensitivity in Cancer (GDSC, previously named CGP), and The Cancer Therapeutics Response Portal (CTRP) cohorts, mainly including antiangiogenic tyrosine kinase inhibitors Axitinib, Nilotinib, Sorafenib, Pazopanib, Masitinib, and Ki8751 (Fig. 7, and Table 2). These findings show that immune checkpoint inhibitors based on *BTNL9* plus antiangiogenic tyrosine kinase inhibitors could be developed as a potential chemotherapy-free combination treatment strategy for LUAD.

**Table 2:** Loss of *BTNL9 expression* may promote drug resistance toward many targeted therapies in CGP, CCLE, and CTRP cohorts

	Drug	Target	t-value	*p*-value
	IOX2	EGLN1	5.91996	4.56E-09
	PHA-793887	CDK9	4.92172	1.02E-06
	OSI-027	MTOR	4.55428	5.99E-06
	Ispinesib	KIF11	4.42265	1.09E-05
	Nilotinib	ABL1	4.3886	1.30E-05
	Axitinib	PDGFRA	4.33456	1.64E-05
	NG25	MAP 4 K2	4.29746	1.92E-05
	Nilotinib	KIT	4.26379	2.26E-05
	BMS345541	IKBKB	4.12048	4.13E-05
	GSK525762A	BRD2	3.92075	9.50E-05
	CAY10603	HDAC6	3.91591	9.69E-05
	TubastatinA	HDAC6	3.89552	0.000105
	GSK525762A	BRD4	3.83187	0.000136
CGP dataset	PHA-793887	CDK1	3.4184	0.000658
	Fluorouracil	TYMS	3.09815	0.002008
	TPCA-1	IKBKB	3.09042	0.002061
	1,256,580–46-7	ALK	3.01459	0.002646
	CAL-101	PIK3CD	2.99145	0.002852
	Belinostat	HDAC6	2.82403	0.004852
	AT7519	CDK9	2.81016	0.00506
	CP-466722	ATM	2.78047	0.005542
	Enzastaurin	PRKCB	2.76951	0.00573
	SB590885	BRAF_V600E.Mutation	2.6446	0.008337
	Vorinostat	HDAC6	2.54112	0.011233
	Nutlin-3	MDM2	−2.94731	0.003295
	Dasatinib	EPHA2	−3.23912	0.001305
	Quizartinib	FLT3	−3.33865	0.000877
	1,173,900–33-8	PIK3CB	−3.55986	0.00039
	Linifanib	FLT3	−5.1822	2.71E-07
	870,483–87-7	CSF1R	−9.60837	7.14E-21
CCLE dataset	Panobinostat	HDAC1	3.29253	0.001066
	abraxane	TUBB	2.83794	0.00473
	Palbociclib	RB1	2.64989	0.008358
	Topotecan	TOP1	2.45344	0.014498
	Sorafenib	FLT3	−4.25026	2.56E-05
CTRP dataset	9-Fluoro-11,17,21-trihydroxy-16-methylpregna-1,4-diene-3,20-dione	NR3C1	5.30047	1.51E-07
	abraxane	TUBB	4.50938	7.51E-06
	Alisertib	AURKB	4.50069	7.83E-06
	PAC-1	CASP3	4.27611	2.16E-05
	660,868–91-7	PLK1	4.21764	2.79E-05
	Gossypol	BCL2	4.19093	3.12E-05
	Decitabine	DNMT1	4.18042	3.24E-05
	722,544–51-6	AURKB	4.14645	3.74E-05
	Etoposide	TOP2B	4.0066	6.76E-05
	3,5-bis(4-methylbenzylidene)piperidin-4-one	USP13	3.92444	9.47E-05
	180,002–83-9	CNR2	3.91953	9.68E-05
	CICLOPIROX	RRM1	3.90668	0.000102
	Dabrafenib	BRAF_V600E.Mutation	3.84863	0.00014
	BRD-K62801835–001–01-0	EZH2	3.80929	0.000151
	BI2536	PLK1	3.69725	0.000233
	Nilotinib	ABL1	3.67464	0.000255
	Cerulenin	HMGCS1	3.66163	0.000268
	TW-37	BCL2	3.585	0.000358
	zebularine	DNMT1	3.57912	0.000366
	Pevonedistat	NAE1	3.55057	0.000409
	BAS02002358	GPER1	3.52883	0.000442
	KU-60019	ATM	3.52842	0.000443
	narciclasine	RHOA	3.47702	0.000536
	Ki8751	PDGFRA	3.43765	0.000619
	4ly1	HDAC1	3.39584	0.000719
	SCHEMBL12182311	EIF4E	3.35831	0.000822
	Belinostat	HDAC1	3.35122	0.000886
	Masitinib	PDGFRA	3.32101	0.000941
	BIBR1532	TERT	3.31812	0.000949
	UNII-UZ77T1VFBM	BIRC5	3.30878	0.000985
	Nutlin-3	MDM2	3.2982	0.001018
	CHEMBL2058177	EIF4E	3.29052	0.001045
	NSC373989	MDM2	3.24218	0.001239
	Imatinib	ABL1	3.22213	0.001327
	Tacrolimus	PPP3CB	3.13945	0.001761
	Olaparib	PARP1	3.07789	0.002159
	SMR001317659	PDE4A	3.03729	0.002467
	Axitinib	PDGFRA	3.02572	0.002563
	Pubchem_92131101	KIF11	3.01909	0.002617
	abraxane	TUBB1	3.01218	0.002678
	GSK461364	PLK1	2.97879	0.002987
	Sorafenib	PDGFRA	2.93438	0.003443
	Nutlin-3	TP53	2.88952	0.003968
	SMR000068650	S1PR2	2.88871	0.003992
	MK-1775	WEE1	2.88827	0.003985
	BRD-K53855319–001–01-2	SIRT1	2.87063	0.004217
	Apicidin	HDAC1	2.7849	0.005485
	TelomeraseInhibitorIX	TERT	2.7825	0.005527
	Pluripotin	MAPK1	2.76988	0.005741
	PHA-793887	CDK1	2.75543	0.005999
	Vorinostat	HDAC1	2.74595	0.006174
	I-BET151	BRD4	2.73756	0.006332
	SCHEMBL12182311	EIF4A2	2.68306	0.007448
	112,522–64-2	HDAC2	2.66828	0.007855
	RG108	DNMT1	2.65008	0.008212
	PF184	IKBKB	2.63052	0.008701
	Pazopanib	PDGFRA	2.63026	0.008704
	JQ-1	BRD4	2.59578	0.009615
	SCHEMBL13833318	HDAC1	2.56094	0.010633
	CAY10603	HDAC6	2.55261	0.010887
	GSK525762A	BRD4	2.51599	0.012071
	4CA-0620	PLK1	2.43094	0.01529
	BCP9000801	MDM2	2.41963	0.015783
	Belinostat	HDAC6	2.37335	0.018128
	Tipifarnib	FNTA	2.36404	0.018536
	prima-1	TP53	2.35318	0.018879
	BMS345541	IKBKB	2.35317	0.018872
	Gemcitabine	RRM1	2.3315	0.019984
	SMR001317659	PDE4B	2.28061	0.022841
	Doxorubicin	TOP2B	2.26369	0.023866
	Rigosertib	PIK3CA	2.22431	0.026433
	3,5-di-tert-butylchalcone	RARA	−2.40858	0.016249
	Dasatinib	EPHA2	−2.95126	0.003261

### Independent predictive power of *BTNL9* based on multivariate analysis

We used the R package “survival V3.2–10” to construct a Cox model, including known important clinical variables for OS, such as TNM stage, primary therapy outcome, and *BTNL9* expression. We also used multivariate analysis to explore whether *BTNL9* expression was an independent OS factor for TCGA-LUAD patients. The results demonstrated that higher *BTNL9* expression significantly (*p* = 0.049) and independently increased OS (HR = 0.67, 95% CI 0.45–0.99) (Table 3).

**Table 3:** Independent predictive power of *BTNL9* based on multivariate analysis

Characteristics	Total(N)	Univariate analysis		Multivariate analysis	
		Hazard ratio (95% CI)	*P* value	Hazard ratio (95% CI)	*P* value
T stage (T3&T4 vs. T1&T2)	501	2.364 (1.621–3.448)	**< 0.001**	1.911 (1.136–3.217)	**0.015**
N stage (N1&N2&N3 vs. N0)	492	2.606 (1.939–3.503)	**< 0.001**	1.863 (1.260–2.755)	**0.002**
M stage (M1 vs. M0)	360	2.111 (1.232–3.616)	**0.007**	1.662 (0.790–3.496)	0.181
Primary therapy outcome (PD&SD vs. CR&PR)	419	2.786 (1.978–3.924)	**< 0.001**	2.951 (1.949–4.468)	**< 0.001**
BTNL9 (High vs. Low)	504	0.686 (0.511–0.921)	**0.012**	0.669 (0.448–0.999)	**0.049**

# *CR* complete response, *PR* partial response, *SD* stable disease, *PD* progressive disease

### Development of a nomogram predicting OS

**Fig. 8:**
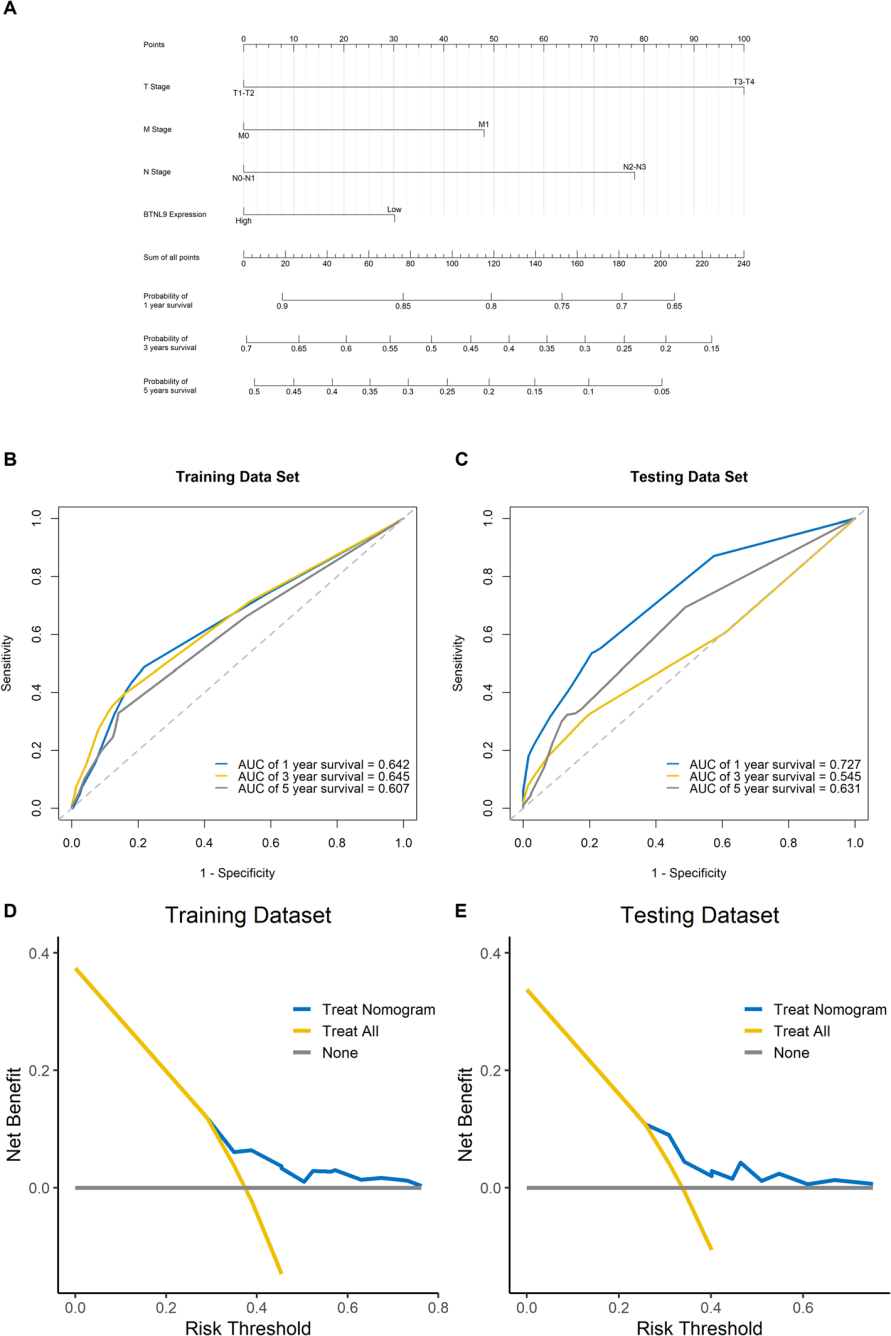
A nomogram predicting the OS for LUAD was constructed. (**A**). A prognostic nomogram for OS with scales for the *BTNL9* and TNM stage was constructed. Validation of the nomogram and clinical usefulness in the training dataset (**B**, **D**) and testing dataset (**C**, **E**)

A nomogram predicting the 1-, 3- and 5- year OS for TCGA-LUAD was constructed based on *BTNL9* expression and TNM stage (Fig. 8A). We built the ROC for the training dataset and the testing dataset and calculated the area under the ROC (AUC) to validate the accuracy of the nomogram. The AUCs for 1-, 3- and 5-year OS were 0.642, 0.645, and 0.607 in the training set (Fig. 8B); 0.727, 0.545, and 0.631 in the testing set (Fig. 8C). These results suggested that the nomogram showed a consistent accuracy in the training and testing dataset. We then conducted decision curve analysis (DCA) to evaluate the clinical usefulness, and the result showed that the nomogram provided an additional benefit compared to the “treat-all” and “treat-none” strategies in both the training and testing dataset (Fig. 8D-E). Finally, to compare the consistency of the model predictions with actual clinical outcomes, calibration curves for 1-year, 3-year, and 5-year OS were created for the training and testing dataset (supplementary Fig. 1A-F). The calibration curves showed consistent agreement between the predicted and observed values for 1-, 3- and 5-year OS.

## Discussion

Immune checkpoint inhibition or adoptive cell therapy has significantly changed the cancer treatment paradigm and has resulted in an age of chemotherapy-free NSCLC [38]. Immune checkpoint inhibitors figure prominently in achieving chemotherapy-free cancer treatment. *BTNs* are immune checkpoints in several cancer types; however, the functions of *BTNs* have not been explored in LUAD. This study shows that *BTNL9* is poorly expressed in LUAD tissues, and its low expression is correlated with a lower probability of 1, 3, 5-year OS based on a nomogram model. In addition, this team explored the mechanisms behind *BTNL9* low expression. This study shows that mutated *p53* results in a significant decrease in *BTNL9* expression (Fig. 2B). Approximately 46% of LUAD patients possess *p53* mutation [39]. Breast cancer exhibits a low expression level of *BTNL9*, which can be targeted to inhibit proliferation and metastasis through the *p53*/CDC25C and *p53*/GADD45 signaling pathway [40]. In addition, *BTNL9* plays a role as a transcriptional modulator through epigenetic regulation and post-transcriptional modification.

DNA methylation is the most common form of DNA modification. It plays a vital role in normal cell physiology, and increased DNA methylation, and loss of demethylation, are observed in different cancer types. DNMTs are implicated in abnormal DNA methylation. Gene body hypermethylation activates oncogenes, and promotion of hypermethylation causes suppression of tumors [41]. GEPIA analysis showed that *BTNL9* and DNMTs correlate significantly with normal lung tissues but not LUAD tumorigenesis (Fig. 4A, B). However, the molecular mechanism of regulation of *BTNL9* by DNMTs in LUAD has not been explored.

miRNA and lncRNA are non-coding RNAs involved in tumor promotion and suppression, depending on the tumor type [41]. A total of 3 miRNAs (hsa-miR-30b-3p, hsa-miR-4709-3p, and hsa-miR-6514-3p) were significantly positively correlated *BTNL9* in LUAD, and their high expression was significantly associated with longer OS. Previous studies reported that hsa-miR-30b-3p plays a role as an antitumor miRNA [42, 43]. Hsu Y-L et al. reported that *BTNL9* acts as a tumor suppressor in LUAD and is regulated by hsa-miR-183-5p; however, the specific regulatory network was not reported [44]. In addition, lncRNA AP001462.6 was shown to bind to *BTNL9*, and the high expression level of this lncRNA was significantly correlated with longer OS in LUAD patients (Fig. 4E).

Moreover, analysis of the protein interaction network of *BTNL9* showed that the interacting proteins played a role in immune regulation, protease hydrolysis, and serine/threonine kinase regulation. Notably, protease hydrolysis is related to ubiquitination and degradation of proteins. Analysis showed that *BTNL9* has a potential E3 recognizing domain binding site (Fig. 4H, I). These findings indicate that *BTNL9* in LUAD may be regulated by DNA methylation and non-coding RNA. In addition, *BTNL9* protein may be held by ubiquitination and degradation after translation.

We performed a GSEA analysis to explore the biological function of *BTNL9* in LUAD. Functional analysis showed low expression levels of *BTNL9* in energy metabolism (oxidative phosphorylation, glycolysis, myc targets v1 [45], and mTORC1 signaling [46]), DNA replication, and protease hydrolysis. Metabolic reprogramming triggers selective gene amplification and a large gene family, which drives cellular functions to promote cancer cell growth and proliferation [45]. The above functions were subsequently verified from a single cell perspective. Findings showed that *BTNL9* was significantly negatively correlated with cancer cell malignant behaviors such as proliferation, invasion, EMT, metastasis, and hypoxia. This result indicates that *BTNL9* may play a role in LUAD tumor suppression.

TME is a potential predictor of response to an immune checkpoint inhibitor. Analysis of the relationship between *BTNL9* and TME showed that the mutation frequency of *BTNL9* in LUAD was about 1.14%, and *BTNL9* was significantly negatively correlated with TMB. Furthermore, *BTNL9* was significantly positively correlated with ImmuneScore and ESTIMATEScore. Previous studies report that high ImmuneScore and ESTIMATEScore are positively associated with a good prognosis of LUAD [47]. This finding shows that *BTNL9* plays an important role in TME immune regulation. Moreover, the correlation between *BTNL9* and TILs showed that *BTNL9* was significantly negatively correlated with tumor purity, and previous studies report that low tumor purity is associated with poor prognosis [48]. Although *BTNL9* was significantly correlated with B, CD4 + T, and macrophages, survival analysis showed that *BTNL9* was only significantly correlated with B cells and DC cells.

DCs act pivotally in shaping innate and adaptive immune responses because they have a unique ability to initiate T-cell responses and promote their differentiation into effector lineages [36]. B cells play antigen presentation, cytotoxicity, and antibody production functions, which are essential in adaptive immunity [49]. TIMER and GEPIA database analysis showed that the expression level of *BTNL9* was not correlated with levels of DCs (cDC1s and cDC2s) in normal adjacent tissues; however, *BTNL9* was significantly associated with levels of DCs (cDC1s and cDC2s) in LUAD tissues (Table 1). cDC1s can migrate to tumor-draining lymph nodes, activate and attract T cells, secrete cytokines, and present antigens in TME, promoting local cytotoxic T cells [50]. cDC2s present antigens to MHC II, activate CD4 + T cells, and effectively polarize TILs into anti-tumor T helper cell 1 (Th1) or Th17 phenotype [51]. The *BTNL9* expression level was not correlated with B cells (naïve B cells) in normal adjacent tissues; however, it was significantly associated with levels of B cells (naïve B cells) in LUAD tissues, except for plasma B cells. This finding implies that *BTNL9* regulates the function of naïve B cells in TME. Previous studies report that naïve B cells are down-regulated in advanced NSCLC and are correlated with poor prognosis [37]. Furthermore, CARE database analysis showed that *BTNL9* expression is associated with effective antiangiogenic tyrosine kinase inhibitors response (Fig. 7, and Table 2). More data are being awaited to confirm this preliminary observation.

Notably, this study had a few limitations. Firstly, our findings are entirely based on public databases using bioinformatics analysis, and therefore further molecular biology experiments should be performed to verify these results. In addition, all findings presented here were developed using Database algorithms. Secondly, *BTNL9* may not be detected by histopathology due to a lack of LUAD tissue samples. Further, OS analyses can’t be performed based on histopathology results. Finally, the study did not verify the role of *BTNL9* in predicting immune responses in LUAD patients due to the lack of clinical cohorts of immunotherapy-treated LUAD patients. Taken together, results and conclusions based entirely on bioinformatics are informative and can lay down the foundations for more robust studies, but they do not replace experimentation.

## Conclusion

In summary, the findings of this study show an association between immune checkpoint *BTNL9* and OS in LUAD patients. Transcriptional regulation and post-transcriptional regulation are potential mechanisms for down-regulating *BTNL9* expression, resulting in more malignant biological characteristics in LUAD. *BTNL9* may modify the TME by enrichment of naïve B cells and DCs and promoting immune response and antiangiogenic tyrosine kinase inhibitors response.

## Supplementary Information

**Additional file 1.**

**Additional file 2.**

**Additional file 3.**

**Additional file 4.**

**Additional file 5. Supplementary Fig. 1** The calibration curves for predicting 1-year, 3-year, and 5-year OS in LUAD. (A, B, and C) Calibration curves for 1, 3, 5-year OS in training dataset; (D, E, and F) Calibration curves for 1, 3, 5-year OS in testing dataset.

## Data Availability

All data in this study were retrieved from public and open-source databases. All databases and its web address were listed below: GEPIA (http://gepia2.cancer-pku.cn/#index), Oncomine (https://www.oncomine.org/), KM plotter (http://www.kmplot.com), UALCAN (http://ualcan.path.uab.edu/index.html), OncoLnc (http://www.oncolnc.org/), TissGDB (https://bioinfo.uth.edu/TissGDB/index.html?csrt=17567836353851237153), TIMER (http://timer.comp-genomics.org/), Sangerbox (http://sangerbox.com/Index), miRMap (https://mirmap.ezlab.org/), TargetScan (http://www.targetscan.org/vert_72/), miRWalk (http://mirwalk.umm.uni-heidelberg.de/), starBase (http://starbase.sysu.edu.cn/panCancer.php), CancerSEA (biocc.hrbmu.edu.cn/CancerSEA/goSearch).

## Abbreviations

LUAD: Lung adenocarcinoma
LUSC: Lung squamous cell carcinoma
NSCLC: Non-small cell lung cancer
*BTN*: Butyrophilin
BTLN: Butyrophilin-like
GEPIA: Gene expression profiling interactive analysis
TIMER: Tumor immune estimation resource
CARE: Computational Analysis of REsistance
GEO: Gene expression omnibus
ECOG: Eastern Cooperative Oncology Group
DNMTs: Methyltransferases
GSEA: Gene set enrichment analysis
scRNA: single-cell RNA
SNV: Single nucleotide mutations
TMB: Tumor mutation burden
CCLE: Cancer Cell Line Encyclopedia
GDSC: Genomics of Drug Sensitivity in Cancer
CTRP: Cancer Therapeutics Response Portal
TILs: Tumor-infiltrating immune cells
TME: Tumor microenvironment

## References

[1] Chen W, Zheng R, Baade PD, Zhang S, Zeng H, Bray F, Jemal A, Yu XQ, He J (2016). Cancer statistics in China, 2015. CA Cancer J Clin.

[2] Bray F, Ferlay J, Soerjomataram I, Siegel RL, Torre LA, Jemal A (2018). Global cancer statistics 2018: GLOBOCAN estimates of incidence and mortality worldwide for 36 cancers in 185 countries. CA Cancer J Clin.

[3] Lu T, Yang X, Huang Y, Zhao M, Li M, Ma K, Yin J, Zhan C, Wang Q (2019). Trends in the incidence, treatment, and survival of patients with lung cancer in the last four decades. Cancer Manag Res.

[4] Garon EB, Hellmann MD, Rizvi NA, Carcereny E, Leighl NB, Ahn MJ, Eder JP, Balmanoukian AS, Aggarwal C, Horn L, Patnaik A, Gubens M, Ramalingam SS, Felip E, Goldman JW, Scalzo C, Jensen E, Kush DA, Hui R (2019). Five-year overall survival for patients with advanced non–small-cell lung Cancer treated with Pembrolizumab: results from the phase I KEYNOTE-001 study. J Clin Oncol.

[5] Davern M, Lysaght J (2020). Cooperation between chemotherapy and immunotherapy in gastroesophageal cancers. Cancer Lett.

[6] Guo Y, Wang AY (2015). Novel Immune Check-Point Regulators in Tolerance Maintenance. Front Immunol.

[7] Abeler-Dörner L, Swamy M, Williams G, Hayday AC, Bas A (2012). Butyrophilins: an emerging family of immune regulators. Trends Immunol.

[8] Malinowska M, Tokarz-Deptuła B, Deptuła W (2017). Butyrophilins: an important new element of resistance. Cent-Eur J Immunol.

[9] Arnett HA, Viney JL (2014). Immune modulation by butyrophilins. Nat Rev Immunol.

[10] Zhou C (2014). Lung cancer molecular epidemiology in China: recent trends. Transl Lung Cancer Res.

[11] Tang Z, Li C, Kang B, Gao G, Li C, Zhang Z (2017). GEPIA: a web server for cancer and normal gene expression profiling and interactive analyses. Nucleic Acids Res.

[12] Clough E, Barrett T (2016). The Gene Expression Omnibus Database. Methods Mol Biol.

[13] Li T, Fu J, Zeng Z, Cohen D, Li J, Chen Q, Li B, Liu XS (2020). TIMER2.0 for analysis of tumor-infiltrating immune cells. Nucleic Acids Res.

[14] Győrffy B, Surowiak P, Budczies J, Lánczky A (2013). Online survival analysis software to assess the prognostic value of biomarkers using transcriptomic data in non-small-cell lung cancer. PLoS One.

[15] Chandrashekar DS, Bashel B, Balasubramanya SAH, Creighton CJ, Ponce-Rodriguez I, Chakravarthi B, Varambally S (2017). UALCAN: A Portal for Facilitating Tumor Subgroup Gene Expression and Survival Analyses. Neoplasia.

[16] Anaya J (2016). OncoLnc: linking TCGA survival data to mRNAs, miRNAs, and lncRNAs. PeerJ Computer Science.

[17] Rhodes DR, Yu J, Shanker K, Deshpande N, Varambally R, Ghosh D, Barrette T, Pandey A, Chinnaiyan AM (2004). ONCOMINE: a cancer microarray database and integrated data-mining platform. Neoplasia.

[18] Kim P, Park A, Han G, Sun H, Jia P, Zhao Z (2017). TissGDB: tissue-specific gene database in cancer. Nucleic Acids Res.

[19] Tang Z, Kang B, Li C, Chen T, Zhang Z (2019). GEPIA2: an enhanced web server for large-scale expression profiling and interactive analysis. Nucleic Acids Res.

[20] Li T, Fan J, Wang B, Traugh N, Chen Q, Liu JS, Li B, Liu XS (2017). TIMER: a web server for comprehensive analysis of tumor-infiltrating immune cells. Cancer Res.

[21] Dai D, Chen B, Feng Y, Wang W, Jiang Y, Huang H, Liu J (2020). Prognostic value of prostaglandin I2 synthase and its correlation with tumor-infiltrating immune cells in lung cancer, ovarian cancer, and gastric cancer. Aging.

[22] Vejnar CE, Blum M, Zdobnov EM (2013). miRmap web: Comprehensive microRNA target prediction online. Nucleic Acid Res.

[23] Agarwal V, Bell GW, Nam JW, Bartel DP (2015). Predicting effective microRNA target sites in mammalian mRNAs. eLife.

[24] Sticht C, De La Torre C, Parveen A, Gretz N (2018). miRWalk: an online resource for prediction of microRNA binding sites. PLoS One.

[25] Li J-H, Liu S, Zhou H, Qu L-H, Yang J-H (2013). starBase v2.0: decoding miRNA-ceRNA, miRNA-ncRNA and protein–RNA interaction networks from large-scale CLIP-Seq data. Nucleic Acids Res.

[26] Jiang P, Lee W, Li X, Johnson C, Liu JS, Brown M, Aster JC, Liu XS (2018). Genome-Scale Signatures of Gene Interaction from Compound Screens Predict Clinical Efficacy of Targeted Cancer Therapies. Cell Syst.

[27] Mizuno H, Kitada K, Nakai K, Sarai A (2009). PrognoScan: a new database for meta-analysis of the prognostic value of genes. BMC Med Genet.

[28] Li Y, Li L, Wang Z, Pan T, Sahni N, Jin X, Wang G, Li J, Zheng X, Zhang Y, Xu J, Yi S, Li X (2018). LncMAP: Pan-cancer atlas of long noncoding RNA-mediated transcriptional network perturbations. Nucleic Acids Res.

[29] Wang P, Li X, Gao Y, Guo Q, Wang Y, Fang Y, Ma X, Zhi H, Zhou D, Shen W (2019). LncACTdb 2.0: an updated database of experimentally supported ceRNA interactions curated from low-and high-throughput experiments. Nucleic Acids Res.

[30] Szklarczyk D, Gable AL, Lyon D, Junge A, Wyder S, Huerta-Cepas J, Simonovic M, Doncheva NT, Morris JH, Bork P, Jensen LJ, Mering C (2018). STRING v11: protein–protein association networks with increased coverage, supporting functional discovery in genome-wide experimental datasets. Nucleic Acids Res.

[31] Shannon P, Markiel A, Ozier O, Baliga NS, Wang JT, Ramage D, Amin N, Schwikowski B, Ideker T (2003). Cytoscape: a software environment for integrated models of biomolecular interaction networks. Genome Res.

[32] Li Y, Xie P, Lu L, Wang J, Diao L, Liu Z, Guo F, He Y, Liu Y, Huang Q, Liang H, Li D, He F (2017). An integrated bioinformatics platform for investigating the human E3 ubiquitin ligase-substrate interaction network. Nat Commun.

[33] Faubert B, Solmonson A, DeBerardinis RJ (2020). Metabolic reprogramming and cancer progression. Science.

[34] Yuan H, Yan M, Zhang G, Liu W, Deng C, Liao G, Xu L, Luo T, Yan H, Long Z, Shi A, Zhao T, Xiao Y, Li X (2018). CancerSEA: a cancer single-cell state atlas. Nucleic Acids Res.

[35] Giraldo NA, Sanchez-Salas R, Peske JD, Vano Y, Becht E, Petitprez F, Validire P, Ingels A, Cathelineau X, Fridman WH, Sautès-Fridman C (2019). The clinical role of the TME in solid cancer. Br J Cancer.

[36] Brown CC, Gudjonson H, Pritykin Y, Deep D, Lavallée V-P, Mendoza A, Fromme R, Mazutis L, Ariyan C, Leslie C (2019). Transcriptional Basis of Mouse and Human Dendritic Cell Heterogeneity. Cell.

[37] Chen J, Tan Y, Sun F, Hou L, Zhang C, Ge T, Yu H, Wu C, Zhu Y, Duan L, Wu L, Song N, Zhang L, Zhang W, Wang D, Chen C, Wu C, Jiang G, Zhang P (2020). Single-cell transcriptome and antigen-immunoglobin analysis reveals the diversity of B cells in non-small cell lung cancer. Genome Biol.

[38] Chu T, Zhong R, Zhong H, Zhang B, Zhang W, Shi C, et al. Phase Ib study of Sintilimab plus Anlotinib as first-line therapy in patients with advanced non-small cell lung Cancer. J Thorac Oncol. 2021;16(4):643-52. 10.1016/j.jtho.2020.11.026. 10.1016/j.jtho.2020.11.026 33524601

[39] Collisson EA, Campbell JD, Brooks AN, Berger AH, Lee W, Chmielecki J, Beer DG, Cope L, Creighton CJ, Danilova L (2014). Comprehensive molecular profiling of lung adenocarcinoma. Nature.

[40] Mo Q, Xu K, Luo C, Zhang Q, Wang L, Ren G (2021). BTNL9 is frequently downregulated and inhibits proliferation and metastasis via the P53/CDC25C and P53/GADD45 pathways in breast cancer. Biochem Biophys Res Commun.

[41] Cheng Y, He C, Wang M, Ma X, Mo F, Yang S, Han J, Wei X (2019). Targeting epigenetic regulators for cancer therapy: mechanisms and advances in clinical trials. Signal Transduct Target Ther.

[42] Zhong K, Chen K, Han L, Li B (2014). MicroRNA-30b/c inhibits non-small cell lung cancer cell proliferation by targeting Rab18. BMC Cancer.

[43] Gao D, Zhou Z, Huang H (2019). miR-30b-3p Inhibits Proliferation and Invasion of Hepatocellular Carcinoma Cells via Suppressing PI3K/Akt Pathway. Front Genet.

[44] Hsu Y-L, Hung J-Y, Lee Y-L, Chen F-W, Chang K-F, Chang W-A, Tsai Y-M, Chong I-W, Kuo P-L (2017). Identification of novel gene expression signature in lung adenocarcinoma by using next-generation sequencing data and bioinformatics analysis. Oncotarget.

[45] Miller DM, Thomas SD, Islam A, Muench D, Sedoris K (2012). C-Myc and cancer metabolism. Clin Cancer Res.

[46] Valvezan AJ, Manning BD (2019). Molecular logic of mTORC1 signalling as a metabolic rheostat. Nat Metab.

[47] Öjlert ÅK, Halvorsen AR, Nebdal D, Lund-Iversen M, Solberg S, Brustugun OT, Lingjaerde OC, Helland Å (2019). The immune microenvironment in non-small cell lung cancer is predictive of prognosis after surgery. Mol Oncol.

[48] Mao Y, Feng Q, Zheng P, Yang L, Liu T, Xu Y, Zhu D, Chang W, Ji M, Ren L, Wei Y, He G, Xu J (2018). Low tumor purity is associated with poor prognosis, heavy mutation burden, and intense immune phenotype in colon cancer. Cancer Manag Res.

[49] Hu X, Zhang J, Wang J, Fu J, Li T, Zheng X, Wang B, Gu S, Jiang P, Fan J, Ying X, Zhang J, Carroll MC, Wucherpfennig KW, Hacohen N, Zhang F, Zhang P, Liu JS, Li B, Liu XS (2019). Landscape of B cell immunity and related immune evasion in human cancers. Nat Genet.

[50] Böttcher JP, Reis e Sousa C (2018). The role of type 1 conventional dendritic cells in Cancer immunity. Trends Cancer.

[51] Perez CR, De Palma M (2019). Engineering dendritic cell vaccines to improve cancer immunotherapy. Nat Commun.

