# Correction to: Butyrophilin-like 9 expression is associated with outcome in lung adenocarcinoma

**DOI:** 10.1186/s12885-021-08874-6

**Published:** 2021-10-26

**Authors:** Weishuang Ma, Jiaming Liang, Junjian Mo, Siyuan Zhang, Ningdong Hu, Dongbo Tian, Zisheng Chen

**Affiliations:** 1 Department of Respiratory Medicine, The Sixth Affiliated Hospital of Guangzhou Medical University Qingyuan People’s Hospital, Qingyuan, China; 2 Zhouxin Community Health Service, Qingcheng District, Qingyuan, China; 3 State Key Laboratory of Respiratory Disease, The First Affiliated Hospital of Guangzhou Medical University, National Clinical Research Center for Respiratory Disease, Guangzhou, China; 4 Department of Thoracic Surgery, The Sixth Affiliated Hospital of Guangzhou Medical University, Qingyuan People’s Hospital, Qingyuan, China

**Correction to: BMC Cancer 21, 1096 (2021)**

**https://doi.org/10.1186/s12885-021-08790-9**

Following publication of the original article [1], the authors identified an error in Fig. 2. The gene BTN3A1 has Chinese text characters, and BTN3A2, BTN3A3, BTNL2, BTNL3, BTNL8, BTNL9, BTNL10, and SKINTL are not marked.

**Fig. 2:**
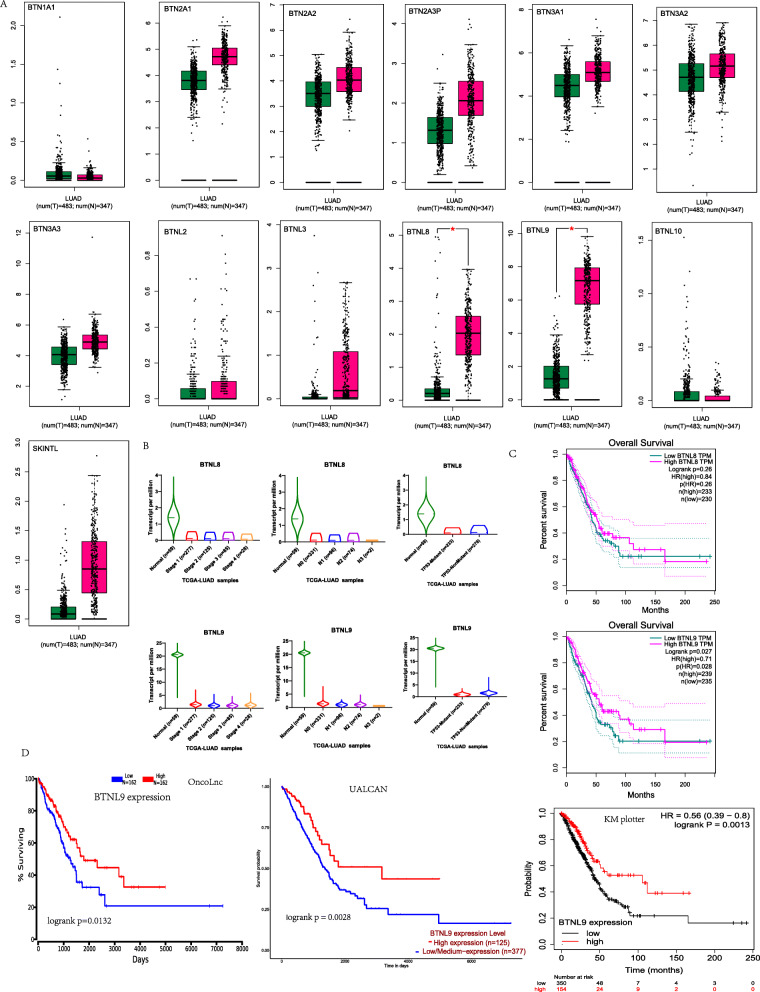
*BTNL9* Expression and prognosis in LUAD. **A** *BTNs* expression panel in LUAD tissues compared with adjacent tissues in GEPIA database. **B** Correlation between *BTNL8* and *BTNL9* expression and LUAD clinical stages, N stage, and *p53* mutation in the UALCAN database (*BTNL8* expression compared with LUAD clinical stages (Normal-vs-Stage1, *P* = 1.01E-04; Normal-vs-Stage2, *P* = 7.08E-03; Normal-vs-Stage3, *P* = 1.36E-04; Normal-vs-Stage4, *P* = 1.78E-08), N stage (Normalvs-N0, *P* = 6.56E-04; Normal-vs-N1, *P* = 8.27E-10; Normal-vs-N2, *P* = 5.96E-04; Normal-vs-N3, *P* = 1.97E-10; N0-vs-N1, *P* = 3.27E-03; N0-vs-N3, *P* = 6.79E-04), and *p53* status (Normal-vs-T P53-Mutant, *P* = 1.27E-06; Normal-vs-T P53-NonMutant, *P* = 1.80E-03). *BTNL9* expression comparison with LUAD clinical stages (Normal-vsStage1, *P* = 1.21E-12; Normal-vs-Stage2, *P* = 3.60E-13; Normal-vs-Stage3, *P* = 2.40E-12; Normal-vs-Stage4, *P* = 2.25E-12), N stage (Normal-vs-N0, *P* = 1.08E-12; Normal-vs-N1, *P* = 1.85E-12; Normal-vs-N2, *P* = 1.02E-12; Normal-vs-N3, *P* = 1.67E-12; N0-vs-N1, *P* = 1.02E-03; N2-vs-N3, *P* = 3.01E-02), and P53 status (Normal-vs-T P53-Mutant, *P* = 1.85E-12; Normal-vs-T P53-NonMutant, *P* = 4.10E-12; T P53-Mutant-vs-T P53-NonMutant, *P* = 1.77E-04)). **C** Correlation between *BTNL8* and *BTNL9* expression with overall survival of LUAD using GEPIA database. **D** Correlation between *BTNL9* expression and LUAD overall survival using OncoLnc, UALCAN, and KM plotter databases. ^*^: *P*-value < 0.05; ^**^: *P*-value < 0.01; ^***^: *P*-value < 0.001

The corrected Fig. 2 is given below and the original article [1] has been corrected.
