# Supplementary material for: Butyrophilin-like 9 expression is associated with outcome in lung adenocarcinoma
Source: BMC Cancer. 2021 Oct 11;21:1096. doi: 10.1186/s12885-021-08790-9 (PMC8507344; doi:10.1186/s12885-021-08790-9)
Supplement: Supplementary file 1 — Additional file 1. [file 12885_2021_8790_MOESM1_ESM.docx]

Supplementary Table 1. Positive results associated with *BTNL9* expression in NSCLC from PrognoScan database

| Dataset | PROBE ID | Endpoint | Number (n) | ln (HR-high / HR-low) | COX P-value | ln (HR) | HR [95% CI-low CI-up] |
| --- | --- | --- | --- | --- | --- | --- | --- |
| GSE31210 | 229985_at | Overall Survival | 204 | -1.2533 | 0.0001 | -0.5701 | 0.57 [0.42 - 0.76] |
|  | 229985_at | Relapse Free Survival | 204 | -1.4294 | 2.03E-07 | -0.5811 | 0.56 [0.45 - 0.70] |
| GSE31210 | 1553279_at | Overall Survival | 204 | -1.7237 | 0.0001 | -0.8315 | 0.44 [0.29 - 0.66] |
|  | 1553279_at | Relapse Free Survival | 204 | -1.3036 | 2.38E-05 | -0.6821 | 0.51 [0.37 - 0.69] |
| GSE31210 | 228434_at | Overall Survival | 204 | -1.1415 | 0.0058 | -0.5335 | 0.59 [0.40 - 0.86] |
|  | 228434_at | Relapse Free Survival | 204 | -1.4818 | 8.06E-07 | -0.7667 | 0.46 [0.34 - 0.63] |
| GSE31210 | 241496_at | Relapse Free Survival | 204 | -1.3868 | 0.0452 | -0.3561 | 0.70 [0.49 - 0.99] |
| GSE3141 | 229985_at | Overall Survival | 111 | -0.8430 | 0.0105 | -0.2989 | 0.74 [0.59 - 0.93] |
