# Supplementary material for: Butyrophilin-like 9 expression is associated with outcome in lung adenocarcinoma
Source: BMC Cancer. 2021 Oct 11;21:1096. doi: 10.1186/s12885-021-08790-9 (PMC8507344; doi:10.1186/s12885-021-08790-9)
Supplement: Supplementary file 2 — Additional file 2. [file 12885_2021_8790_MOESM2_ESM.docx]

Supplementary Table 2. *BTNL9* significant low expression in cancers vs. normal tissue in Oncomine database

| Cohort | Cancer type | Sample (n) | Rank (%) | Fold change | P value | PMID |
| --- | --- | --- | --- | --- | --- | --- |
| TCGA Breast cancer | Mixed Lobular and Ductal Breast Carcinoma | 68 | 1 | -3.974 | 7.26E-15 | TCGA |
|  | Male Breast Carcinoma | 64 | 1 | -3.280 | 5.74E-20 | TCGA |
|  | Invasive Lobular Breast Carcinoma | 97 | 1 | -3.747 | 3.21E-18 | TCGA |
|  | Invasive Breast Carcinoma | 137 | 1 | -5.769 | 5.34E-30 | TCGA |
|  | Invasive Ductal Breast Carcinoma | 450 | 2 | -8.068 | 2.52E-37 | TCGA |
|  | Mucinous Breast Carcinoma | 65 | 3 | -7.675 | 2.34E-5 | TCGA |
| Radvanyi Breast cancer | Invasive Ductal Breast Carcinoma | 30 | 1 | -10.067 | 3.74E-6 | 16043716 |
| Curtis Breast cancer | Breast Carcinoma | 158 | 1 | -3.171 | 1.72E-23 | 22522925 |
|  | Mucinous Breast Carcinoma | 190 | 1 | -3.032 | 2.54E-40 | 22522926 |
|  | Invasive Breast Carcinoma | 165 | 1 | -3.103 | 6.03E-27 | 22522927 |
|  | Ductal Breast Carcinoma in Situ | 154 | 1 | -2.932 | 3.99E-14 | 22522928 |
|  | Medullary Breast Carcinoma | 176 | 1 | -3.155 | 1.50E-40 | 22522929 |
|  | Tubular Breast Carcinoma | 211 | 1 | -3.088 | 5.43E-41 | 22522930 |
|  | Invasive Ductal and Invasive Lobular Breast Carcinoma | 234 | 2 | -2.999 | 3.60E-40 | 22522931 |
|  | Invasive Lobular Breast Carcinoma | 292 | 3 | -2.913 | 4.85E-39 | 22522932 |
|  | Invasive Ductal Breast Carcinoma | 1700 | 7 | -3.149 | 8.16E-42 | 22522933 |
| Richardson Breast cancer | Ductal Breast Carcinoma | 47 | 6 | -7.434 | 5.53E-06 | 16473279 |
| Skrzypczak Colorectal cancer | Colon Adenoma | 15 | 2 | -2.475 | 9.95E-8 | 20957034 |
| Yusenko Renal cancer | Papillary Renal Cell Carcinoma | 5 | 2 | -4.346 | 1.52E-5 | 19445733 |
| Hou Lung cancer | Lung Adenocarcinoma | 110 | 1 | -9.102 | 1.02E-24 | 20421987 |
|  | Squamous Cell Lung Carcinoma | 92 | 2 | -11.299 | 1.40E-21 | 20421988 |
|  | Large Cell Lung Carcinoma | 84 | 4 | -11.144 | 4.70E-11 | 20421989 |
| Okayama Lung cancer | Lung Adenocarcinoma | 246 | 3 | -12.000 | 3.77E-14 | 22080568 |
| Crabtree Uterus cancer | Uterine Corpus Leiomyoma | 77 | 3 | -2.178 | 9.47E-6 | 19622772 |
