# Supplementary material for: Butyrophilin-like 9 expression is associated with outcome in lung adenocarcinoma
Source: BMC Cancer. 2021 Oct 11;21:1096. doi: 10.1186/s12885-021-08790-9 (PMC8507344; doi:10.1186/s12885-021-08790-9)
Supplement: Supplementary file 3 — Additional file 3. [file 12885_2021_8790_MOESM3_ESM.docx]

Supplementary Table 3. lncRNA-TF-gene triplets of the *BTNL9* in LncMAP database

| Cancer Type | LncRNA ID | LncRNA Symbol | TF ID | TF Symbol | Gene ID | Gene Symbol | P-Value Discovery | P-Value Validation |
| --- | --- | --- | --- | --- | --- | --- | --- | --- |
| LUAD | ENSG00000271978 | RP11-428J1.4 | ENSG00000170345 | FOS | ENSG00000165810 | BTNL9 | 0.0115 | 0.955 |
| LUAD | ENSG00000270949 | RP11-288H12.4 | ENSG00000170345 | FOS | ENSG00000165810 | BTNL9 | 0.013 | 0.61 |
| LUAD | ENSG00000271914 | RP4-789D17.5 | ENSG00000170345 | FOS | ENSG00000165810 | BTNL9 | 0.0153 | 0.661 |
| LUAD | ENSG00000238198 | RP11-31F15.2 | ENSG00000177606 | JUN | ENSG00000165810 | BTNL9 | 0.0169 | 0.217 |
| LUAD | ENSG00000265787 | CYP4F35P | ENSG00000177606 | JUN | ENSG00000165810 | BTNL9 | 0.0174 | 0.335 |
| LUAD | ENSG00000273290 | CTC-297N7.8 | ENSG00000170345 | FOS | ENSG00000165810 | BTNL9 | 0.0215 | 0.79 |
| LUAD | ENSG00000231125 | AF129075.5 | ENSG00000170345 | FOS | ENSG00000165810 | BTNL9 | 0.0237 | 0.653 |
| LUAD | ENSG00000260583 | LINC00515 | ENSG00000170345 | FOS | ENSG00000165810 | BTNL9 | 0.0238 | 0.856 |
| LUAD | ENSG00000269038 | AP001462.6 | ENSG00000170345 | FOS | ENSG00000165810 | BTNL9 | 0.0262 | 0.717 |
| LUAD | ENSG00000261141 | RP11-303E16.5 | ENSG00000170345 | FOS | ENSG00000165810 | BTNL9 | 0.0327 | 0.6 |
| LUAD | ENSG00000217576 | RP11-248G5.8 | ENSG00000170345 | FOS | ENSG00000165810 | BTNL9 | 0.0033 | 0.646 |
| LUAD | ENSG00000254295 | CTC-308K20.2 | ENSG00000177606 | JUN | ENSG00000165810 | BTNL9 | 0.004 | 0.793 |
| LUAD | ENSG00000227960 | RP5-837I24.6 | ENSG00000170345 | FOS | ENSG00000165810 | BTNL9 | 0.044 | 0.686 |
| LUAD | ENSG00000249859 | PVT1 | ENSG00000170345 | FOS | ENSG00000165810 | BTNL9 | 0.0072 | 0.995 |
| LUAD | ENSG00000272555 | RP11-459I19.1 | ENSG00000170345 | FOS | ENSG00000165810 | BTNL9 | 0.0073 | 0.954 |
| LUAD | ENSG00000227954 | RP3-323P13.2 | ENSG00000177606 | JUN | ENSG00000165810 | BTNL9 | 0.0083 | 0.0707 |
| LUAD | ENSG00000260917 | RP11-57H14.4 | ENSG00000170345 | FOS | ENSG00000165810 | BTNL9 | 0.0083 | 0.818 |
| LUAD | ENSG00000272143 | FGF14-AS2 | ENSG00000170345 | FOS | ENSG00000165810 | BTNL9 | 0.0088 | 0.501 |
