# Supplementary material for: Butyrophilin-like 9 expression is associated with outcome in lung adenocarcinoma
Source: BMC Cancer. 2021 Oct 11;21:1096. doi: 10.1186/s12885-021-08790-9 (PMC8507344; doi:10.1186/s12885-021-08790-9)
Supplement: Supplementary file 4 — Additional file 4. [file 12885_2021_8790_MOESM4_ESM.docx]

Supplementary Table 4. Predicted E3 ligases of *BTNL9* in ubibrowser database

| Gene symbol | Gene description | Score | Confidence level |
| --- | --- | --- | --- |
| MARCH8 | E3 ubiquitin-protein ligase MARCH8 | 0.805 | High |
| TRIM25 | E3 ubiquitin/ISG15 ligase TRIM25 | 0.805 | High |
| MARCH1 | E3 ubiquitin-protein ligase MARCH1 | 0.740 | Middle |
| MARCH9 | E3 ubiquitin-protein ligase MARCH9 | 0.740 | Middle |
| TRIM27 | Zinc finger protein RFP | 0.733 | Middle |
| MARCH4 | E3 ubiquitin-protein ligase MARCH4 | 0.716 | Middle |
| MARCH11 | E3 ubiquitin-protein ligase MARCH11 | 0.716 | Middle |
| SYVN1 | E3 ubiquitin-protein ligase synoviolin | 0.714 | Middle |
| CBL | E3 ubiquitin-protein ligase CBL | 0.703 | Middle |
| MARCH7 | E3 ubiquitin-protein ligase MARCH7 | 0.696 | Middle |
| PML | Protein PML | 0.664 | Low |
| BFAR | Bifunctional apoptosis regulator | 0.664 | Low |
| BTRC | F-box/WD repeat-containing protein 1A | 0.651 | Low |
| TRIM23 | E3 ubiquitin-protein ligase TRIM23 | 0.651 | Low |
| MARCH3 | E3 ubiquitin-protein ligase MARCH3 | 0.646 | Low |
