# Supplementary figures and images for: Butyrophilin-like 9 expression is associated with outcome in lung adenocarcinoma
Source: BMC Cancer. 2021 Oct 11;21:1096. doi: 10.1186/s12885-021-08790-9 (PMC8507344; doi:10.1186/s12885-021-08790-9)

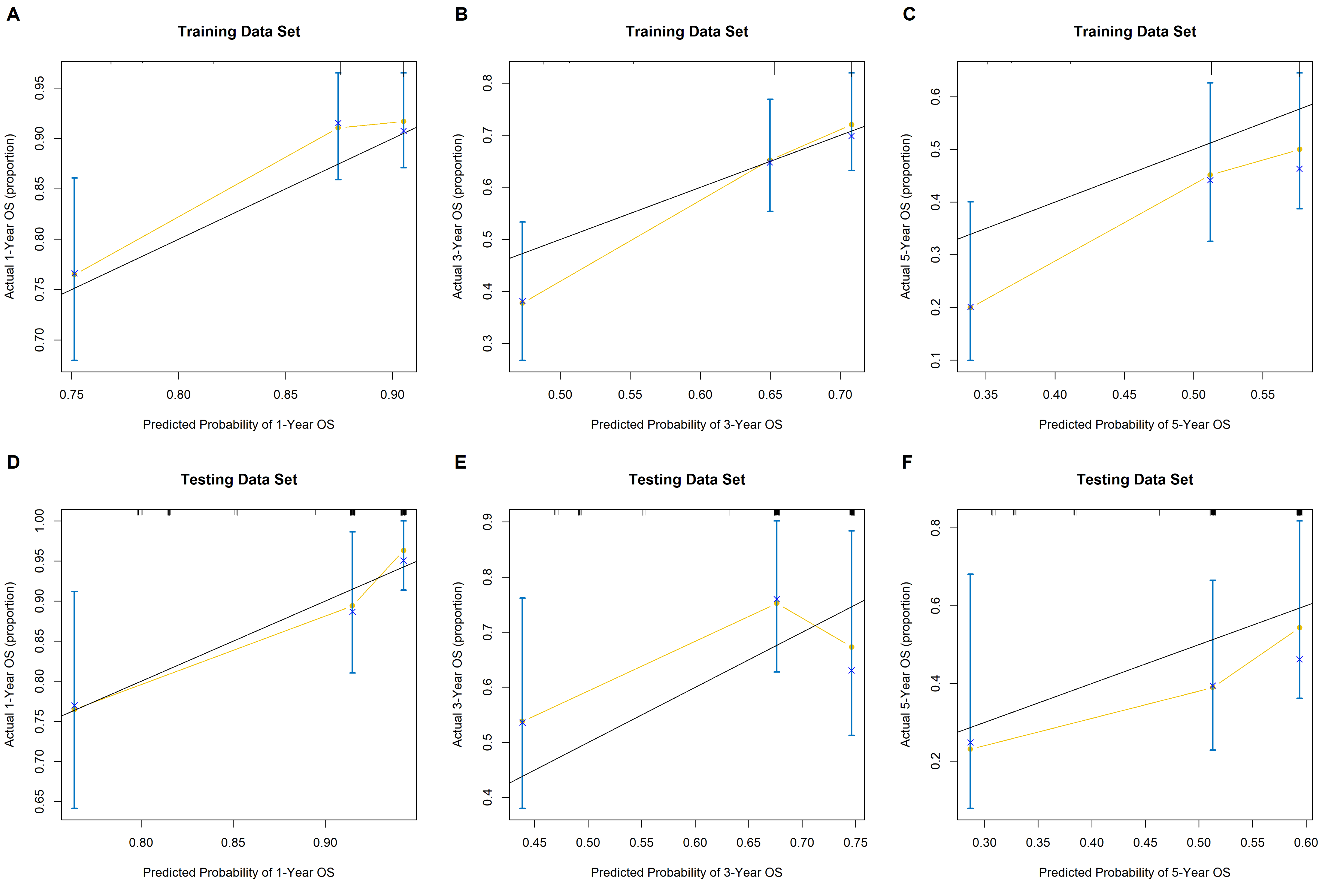

Supplement: Supplementary file 5 — Additional file 5. Supplementary Fig. 1 The calibration curves for predicting 1-year, 3-year, and 5-year OS in LUAD. (A, B, and C) Calibration curves for 1, 3, 5-year OS in training dataset; (D, E, and F) Calibration curves for 1, 3, 5-year OS in testing dataset. [file 12885_2021_8790_MOESM5_ESM.tiff]
